# Gene Network Construction from Microarray Data Identifies a Key Network Module and Several Candidate Hub Genes in Age-Associated Spatial Learning Impairment

**DOI:** 10.3389/fnsys.2017.00075

**Published:** 2017-10-10

**Authors:** Raihan Uddin, Shiva M. Singh

**Affiliations:** Department of Biology, University of Western Ontario, London, ON, Canada

**Keywords:** microarray, spatial learning, learning impairment, brains, data integration, mathematical modeling, gene networks, WGCNA

## Abstract

As humans age many suffer from a decrease in normal brain functions including spatial learning impairments. This study aimed to better understand the molecular mechanisms in age-associated spatial learning impairment (ASLI). We used a mathematical modeling approach implemented in Weighted Gene Co-expression Network Analysis (WGCNA) to create and compare gene network models of young (learning unimpaired) and aged (predominantly learning impaired) brains from a set of exploratory datasets in rats in the context of ASLI. The major goal was to overcome some of the limitations previously observed in the traditional meta- and pathway analysis using these data, and identify novel ASLI related genes and their networks based on co-expression relationship of genes. This analysis identified a set of network modules in the young, each of which is highly enriched with genes functioning in broad but distinct GO functional categories or biological pathways. Interestingly, the analysis pointed to a single module that was highly enriched with genes functioning in “learning and memory” related functions and pathways. Subsequent differential network analysis of this “learning and memory” module in the aged (predominantly learning impaired) rats compared to the young learning unimpaired rats allowed us to identify a set of novel ASLI candidate hub genes. Some of these genes show significant repeatability in networks generated from independent young and aged validation datasets. These hub genes are highly co-expressed with other genes in the network, which not only show differential expression but also differential co-expression and differential connectivity across age and learning impairment. The known function of these hub genes indicate that they play key roles in critical pathways, including kinase and phosphatase signaling, in functions related to various ion channels, and in maintaining neuronal integrity relating to synaptic plasticity and memory formation. Taken together, they provide a new insight and generate new hypotheses into the molecular mechanisms responsible for age associated learning impairment, including spatial learning.

## Introduction

One of the most significant effects of aging is the decrease in normal brain functions, particularly, cognition and memory. The incidence of cognitive impairments, including normal age-associated spatial learning impairment (ASLI), has risen dramatically in past decades due to increasing human longevity (Burger et al., 2007; Peleg et al., 2010; Glorioso et al., 2011). As such trends are expected to continue, it has become imperative to better understand the underlying molecular biology and genetics of ASLI. Towards that goal, in a previous meta-analysis study (Uddin and Singh, 2013), we integrated several microarray gene-expression data generated from independent studies in the context of ASLI in the hippocampus in rats. The data represented young rats that were learning unimpaired and aged rats that were learning impaired and unimpaired. The carefully designed original studies investigated spatial learning tasks in young (3–6 months old) and aged (24–26 months old) animals using the Morris Water Maze as the training and assessment protocol. All experimental young animals demonstrated learning unimpairment. While the majority of the experimental aged animals demonstrated learning impairment, some demonstrated learning unimpairment. Since, hippocampus in the brain is integral to memory function including spatial memory both in humans and in rodents (Morris et al., 1982; Burgess, 2002), microarray gene-expression data were generated using the hippocampus tissue. These datasets allowed us to assess a combined gene expression changes related to aging, as well as ASLI in rats across multiple studies (Uddin and Singh, 2013). We used traditional methods such as differential expression analysis, followed by functional and pathway analysis using the Ingenuity Pathway Analysis (IPA) software^1^, to identify ASLI genes and networks. Though our meta-analysis identified a number of significant differentially expressed genes and networks across age or across ASLI in several interesting biological categories, however, the results highlighted some limitations in such traditional analyses. One of the limitations is that gene networks and regulatory interactions among the genes in these networks are modeled based on current biological knowledge only. For example, IPA pathway or similar knowledge base analysis can only model gene networks based on information that is available in the literature. Therefore, such analyses are unable to fully utilize the gene transcript expression information captured by the microarray data. Another limitation is that they are not able to identify a single network that could be solely associated with ASLI, as we previously observed that the candidate ASLI genes were all scattered in different networks (Uddin and Singh, 2013). Finally, there is no prioritization of molecules within the knowledge-based network models of affected pathways. As a result, to overcome the above limitations, mathematical modeling of gene networks from large scale gene-expression data is becoming a popular alternative choice in the network discovery process, and has proven highly useful in recent years (Friedman et al., 2000; Margolin et al., 2006; Opgen-Rhein and Strimmer, 2007; Langfelder and Horvath, 2008; Ideker and Bandyopadhyay, 2010). Particularly, the correlation-based modeling method implemented in WGCNA (Zhang and Horvath, 2005) has gained a lot of popularity (Fuller et al., 2007; Oldham et al., 2008; Mason et al., 2009; Plaisier et al., 2009; Miller et al., 2010; Levine et al., 2013; Fontenot and Konopka, 2014; Rickabaugh et al., 2015; Ye and Liu, 2015).

Numerous studies have applied gene co-expression network analysis using WGCNA to associate co-expression modules with brain and psychiatric diseases (Oldham et al., 2006; Miller et al., 2008; de Jong et al., 2010; Torkamani et al., 2010; Voineagu et al., 2011). However, no study investigating gene network modeling in ASLI appears in the literature. Therefore, we felt it necessary to initiate such a modeling to explore and identify key functional modules and gene hubs in the context of ASLI. Here, we performed a co-expression network analysis (using WGCNA) as a follow up to our previous study (Uddin and Singh, 2013) using the same datasets. The specific goals in this study were to create gene network models from a set of exploratory datasets, separately for aged (predominantly learning impaired) and young (learning unimpaired) samples; to perform a differential network analysis between these aged and young networks; and to evaluate results (significant functional modules and hub genes) by comparing them against a set of validation datasets.

This analysis has identified several reproducible network modules each highly significant with genes functioning in specific biological functional categories (Uddin, 2015). It identifies a “learning and memory” specific module containing many potential key ASLI hub genes, some of which were also identified (but not prioritized) in the meta-analysis. Many of these candidate hub genes not only show differential co-expression between young and aged networks, but are also reproducible in independent datasets. Functions of these ASLI hub genes link a different set of mechanisms to learning and memory formation, which meta-analysis was unable to detect. Future follow up research can help further understand their potential molecular mechanisms underlying complex behavioral traits such as cognitive impairments including ASLI. Modern meta- and network approaches as implemented in this study can be applied to any large-scale dataset to identify potential key molecules and networks and thus generate new hypotheses.

## Materials and Methods

### Data Selection for Network Analysis

For this study, we have selected five microarray datasets referred here as BL (Blalock et al., 2003), B7 (Burger et al., 2007), R7 (Rowe et al., 2007), B8 (Burger et al., 2008) and K9 (Kadish et al., 2009). They consist of a total of 287 arrays and used two different Affymetrix chip types, RG_U34a and RAE230a. The data represented young rats that were learning unimpaired and aged rats majority of which were learning impaired with some learning unimpaired animals. The BL and K9 studies were similar in design where only the unimpaired young and impaired aged animals were considered for comparison. The B7, R7 and B8 studies were similar in design where both young and aged groups contained controls animals that were learning impaired, e.g., cage controls, stress controls and controls for visual impairment. These datasets were quality checked and normalized using RMA methods (Bolstad et al., 2003; Gautier et al., 2004), had outliers removed and batch effect adjusted using age and spatial learning impairment as covariates (Uddin and Singh, 2013). For each dataset, aged and young samples were separated and assessed further for the presence of array outliers (Supplementary Table S1 and Supplementary Figures S1–S6). Since the WGCNA network construction method is correlation based, before proceeding with network analysis it was made sure that the correlations between genes in each dataset were reasonable as suggested in the literature (Miller et al., 2010). This was done by calculating Pearson’s correlations between the expression levels of each pair of genes in the aged or young preprocessed datasets and by plotting the correlation values in histogram plots (Supplementary Figure S7). All data preparation steps including WGCNA, GO, and statistical analyses were performed in R using appropriate software packages.

### Co-Expression Network Analysis

Using the preprocessed transformed data (genes in columns and samples in rows), gene networks were constructed for aged and young using the WGCNA R package (Zhang and Horvath, 2005; Langfelder and Horvath, 2008) following a slightly modified protocol based on the approaches previously described (Oldham et al., 2006, 2008; Miller et al., 2008, 2010). The overall network analysis process for a single dataset is described below.

### Creating an Adjacency (Connection Strength) Matrix

A weighted correlation between two genes represents connection strength between the genes in a network. For each dataset, a network adjacency or connection strength matrix (network data) was created by taking the signed correlations of the gene expression values between each pair of genes raised to a power of beta. Beta is the weight, a soft threshold, and was determined in advance in such a way so that the resulting network follows approximate scale free topology. The values in the diagonal (self-correlation) were converted to zero.

### Filtering Out Genes with Very Low Connectivity

To save computational time, genes were filtered out from a network adjacency matrix based on their connectivity (i.e., only genes with above average median connectivity were kept for network analysis). The overall connectivity for each gene (denoted by *k*) is the sum of connection strengths (weighted correlation) between that gene and all other genes in the network. It is scaled to lie between 0 and 1 and represents how strongly a gene is connected to all other genes in the network.

### Creating and Visualizing Network Modules

Following filtering an adjacency matrix contained genes with reasonably high network connectivity. This adjacency matrix was used to determine a network topological overlap, construct a hierarchical clustering dendrogram of 1—topological overlap, determine network modules using a hybrid tree-cutting algorithm, and to visualize network modules. In a co-expression network, an edge between two genes (nodes) represents a co-expression relationship. For each dataset or module a network interaction file was created from its adjacency matrix, and used in Cytoscape for visualization and analysis.

Network analysis often results in a large number of modules. It is sometimes useful to reduce the number of modules by merging those whose expression profiles are very similar. This was accomplished by merging modules whose member genes were highly co-expressed. To calculate the co-expression similarity of entire modules, their module eigengenes were calculated. The module eigengene is defined as the first principal component of a given module. It can be considered as a representative of the gene expression profiles in a module. The module eigengenes were clustered on their consensus correlation, which was the minimum correlation across the two sets.

### Exploring the Functional Significance of Modules

A list of genes belonging to each network module was exported to tab delimited text files along with all necessary information. For each module there were two files, the first file contained a list of genes with their gene symbols, mean expression, module names, and intra-modular connectivity. This file was used for GO analysis using The Database for Annotation, Visualization and Integrated Discovery (DAVID)^2^ (Huang et al., 2007, 2009a,b). The second file contained co-expression interaction information between each pair of genes in a module along with the topological overlap and correlation information. This interaction file was used for network visualization and analysis.

GO functional Annotation Clustering analysis was performed through DAVID web-services using the gene list for each young network module. In this research, DAVID web-services were accessed programmatically by using an R package called RDAVIDWebService (Fresno and Fernández, 2013). Since gene symbols can be confusing and often fail to produce a perfect match, the corresponding affymetrix IDs were used to query the DAVID database. GO functional annotation information was obtained for all modules in the young and the aged categories.

In DAVID, for each functional cluster an enrichment score is calculated. This enrichment score is the geometric mean (in −log scale) of the *p*-values of all member annotation terms and is used to rank their biological significance (Huang et al., 2009b). Thus, the top ranked annotation clusters will most likely have consistently lower *p*-values for their annotation members. The significance of a gene-enrichment *p*-values for each annotation term is calculated based on a modified Fisher exact test method known as the EASE score (Hosack et al., 2003). The default threshold of the EASE score was set at 0.1.

### Validating Network Modules

Network modules for young and aged were compared across studies and platforms for their repeatability using the statistics implemented in WGCNA software package. This was done in two ways: (a) module preservation; and (b) module overlap.

#### Module Preservation

Module preservation statistics (Zhang and Horvath, 2005; Miller et al., 2010; Langfelder et al., 2011) can qualitatively and quantitatively measure network preservation at the module level. As a qualitative assessment, the gene module assignment from one network was mapped on the same genes in the second network. The results were then plotted in a dendrogram, which offers a visual mean to qualitatively assess preservation. Quantitative measure of network preservation assesses how well a module in one study is preserved in another study using a number of statistics. Module preservation was estimated quantitatively between the young and the aged networks in different datasets. In all comparisons, the R7 top most connected genes, their transcription profiles, and their module assignments were used as a reference.

#### Module Overlap

Comparing networks by calculating module overlap allows one to determine whether a module that was found in one dataset can also be found in another dataset (Miller et al., 2010; Horvath, 2011). Fisher’s exact test is used to calculate a *p*-value of significance of pair-wise module overlap. In this research, module overlaps were calculated along with their significance of overlaps between the young modules and between the aged modules in different datasets following the approach described in Oldham et al. (2008). In brief, top most connectivity genes common between a network from R7 (exploratory set) aged (or young) and another aged (or young) network from a validation set were selected. Next, the module labels between the two networks were matched. The purpose was to see which modules in one network contain a significant number of overlapping genes with modules in the second network. Next, module labels were reassigned in the second network such that corresponding modules were assigned the same color label. After matching labels between the modules in exploratory and validation networks, their percentage overlaps and significance *p*-values were calculated.

### Differential Network Analysis of Young vs. Aged

Differential network analysis allows one to compare two different networks side by side, for example, between a control and a disease network. Networks for several interesting modules identified in this research were visualized side by side between the young and aged groups using Cytoscape and compared for their differential co-expression.

### Identifying and Validating Hub Genes

Top hub genes were identified by using module eigengene-based connectivity or *k*_ME_ values in both the young (learning unimpaired) and aged (predominantly learning impaired) networks. Module eigengene-based connectivity *k*_ME_, also known as module membership, is calculated for each gene. It is defined by correlating each gene’s expression profile with the module eigengene of a given module (Zhang and Horvath, 2005; Langfelder and Horvath, 2008; Miller et al., 2010). Hub genes were validated by assessing their repeatability in networks constructed from independent datasets and by investigating their functions in relevant pathways. In addition, expression patterns of selected hub genes were verified using meta-analysis and forest plots.

### Repeatability

Repeatability of the candidate hub genes were assessed as follows. For each module, hub genes identified in the exploratory (R7) networks were checked if they are also identified as hub genes in the validation networks (e.g., B8, K9, or B7) with high *k*_ME_ values as well as with *t*-test *p*-values ≤ 0.05 (between two networks at a time e.g., one exploratory and one validation). In cases where a module from an exploratory network matched to multiple modules in a validation network, genes from multiple significant modules in the validation network were combined together and then compared to the hub genes in the exploratory network module.

### Literature Search

Literature searches were performed using PubMed to explore characteristics and functions of selected ASLI candidate hub genes and their relationship to learning and memory formation.

## Results

In order to model, explore and identify ASLI genes and their networks, this analysis followed a detailed and through investigation that included the identification of GO enriched significant functional modules and hub genes, as well as validation of results using independent datasets. The results are described below.

### Data Selection for Network Analysis

Based on the quality of data and number of samples (Supplementary Table S1), R7 aged (R7-A) and young (R7-Y) datasets were chosen as the exploratory datasets; B8 young (B8-Y), K9 young (K9-Y), B7 aged (B7-A), and B8 aged (B8-A) datasets were chosen as the validation datasets (Table 1). After preprocessing, other groups did not have sufficient number of samples for WGCNA, they were excluded from this analysis. The networks were constructed for each of the aged and young datasets separately (i.e., B7-A, B8-Y, B8-A, K9-Y, R7-Y, and R7-A). However, GO based functional analysis and visualization was done only for the networks from R7 young and aged exploratory datasets, and the results were validated independently in networks constructed from the validation datasets. All datasets combined, this research included 65 young rat samples that were identified as learning unimpaired, 66 aged samples that were identified as learning impaired, and 29 aged samples that were identified as learning unimpaired.

**Table 1:** Datasets selected for network analysis.

Dataset	Number of young sample	Exploratory/Validation (Young)	Number of aged sample	Exploratory/Validation (Aged)
B7 (Burger et al., 2007)			28	Validation
R7 (Rowe et al., 2007)	19	Exploratory	27	Exploratory
B8 (Burger et al., 2008)	18	Validation	28	Validation
K9 (Kadish et al., 2009)	18	Validation		

### Determining the Weights or Soft Power Beta

Based on the scale free topology model fit analysis the soft-threshold power for both R7-Y and R7-A was determined to be 6. This power also results in an approximate straight line relationship in the scale-free topology plots (Supplementary Figures S8, S9). Performing similar analyses, the soft powers for B8-Y and B8-A were determined to be 10 and 8, respectively. For the B7-A dataset, the soft power was 9 and for K9-Y it was 10.

### Creating Adjacency (Connection Strength) Matrices

The genes that remained after preprocessing (Supplementary Table S2) were used to calculate the signed Pearson correlation coefficients for all pairwise comparisons of gene-expression values across all young and aged samples. The correlation matrix for each group was then transformed into a matrix of connection strengths (i.e., an “adjacency” matrix) using a soft power beta as determined above. This resulted in a network adjacency matrix for each dataset, for example, for R7 it generated an 8053 × 8053 matrix.

### Filtering Out Genes with Very Low Connectivity

First, connectivity value for each gene was calculated from the adjacency matrix. Next for each dataset, the average median connectivity *k*_med_ was used as a cut-off value to filter out genes with very low connectivity. For R7-Y *k*_med_ was 0.46 and for R7-A *k*_med_ was 0.54. We selected the average *k*_med_ = 0.5 as the minimum connectivity cut-off, which removed 2379 genes, leaving 5674 high connectivity genes for the R7 network analysis (Supplementary Table S2). For B8 and K9 the median *k*_med_ was 0.4 and 0.35, which resulted in 5202 and 4796 high connectivity genes, respectively. The number of B7 genes was already low and close to the numbers of other filtered datasets. So, in order to prevent information loss no filtering was done on these B7 genes.

### Creating and Visualizing Networks and Modules

A major goal of gene correlation network analysis is to identify groups of highly interconnected genes (Zhang and Horvath, 2005; Oldham et al., 2006) termed as modules. The expression profiles of genes in a module are highly correlated across the samples. In a co-expression network, modules are identified by searching for genes with similar patterns of connection strengths to other genes, or genes with high topological overlap. The topological overlap values are calculated using the adjacency and connectivity values, which determine which genes will be in which module and form a network. The values range between 1 and 0 representing maximum and minimum interconnectedness. The module identification method in WGCNA is based on using a node dissimilarity measure in conjunction with a clustering method. Since the topological overlap matrix is non-negative and symmetric, it is turned into a dissimilarity measure by subtracting from 1. Genes are hierarchically clustered using the average linkage method, taking 1-topological overlap as the distance measure and modules are determined by choosing a height cutoff for the resulting dendrogram. In the dendrogram, discrete branches of the tree correspond to modules of co-expressed genes. Following these steps, gene network modules for the young and aged samples were identified separately for each dataset using the filtered weighted correlation matrices as prepared above.

**Figure 1:**
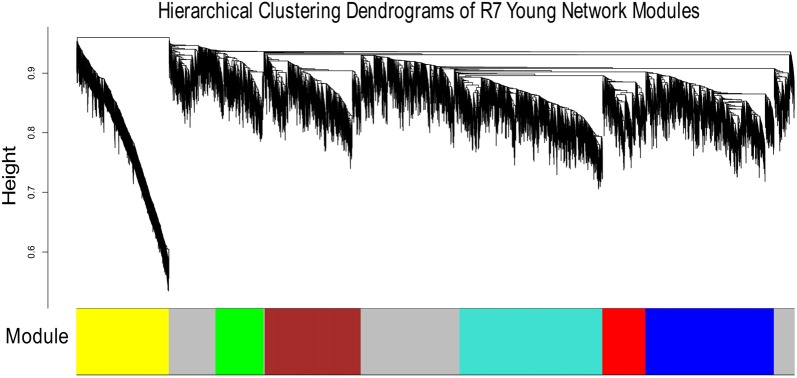
Hierarchical clustering dendrogram of topological overlaps of R7-Y genes. The cut-tree hybrid method was used to pick a height cut-off and to identify modules, which are shown in the panel below the dendrogram. Each module is labeled with a unique color for easy visualization and understanding.

**Figure 2:**
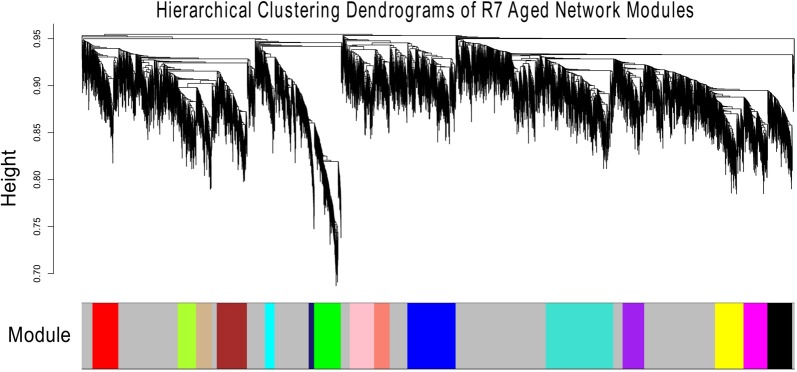
Hierarchical clustering dendrogram of topological overlaps of R7-A genes. The cut-tree hybrid method was used to pick a height cut-off and to identify modules, which are shown in the panel below the dendrogram. Each module is labeled with a unique color for easy visualization and understanding.

Figures 1, 2 show the hierarchical dendrograms of topological overlaps for the 5674 genes in R7-Y and R7-A, respectively. There are several height cut-off algorithms implemented in the WGCNA R package. In this research, the cut-tree hybrid method was chosen to pick a height cut-off and to identify modules, which are shown in the panel below the dendrograms. The default lowest cut-off resulted in six modules in the young network and 15 modules in the aged network. Each module is labeled with a unique color (except gray) for easy visualization and understanding. The color gray is preserved for genes that do not belong to any module.

**Figure 3:**
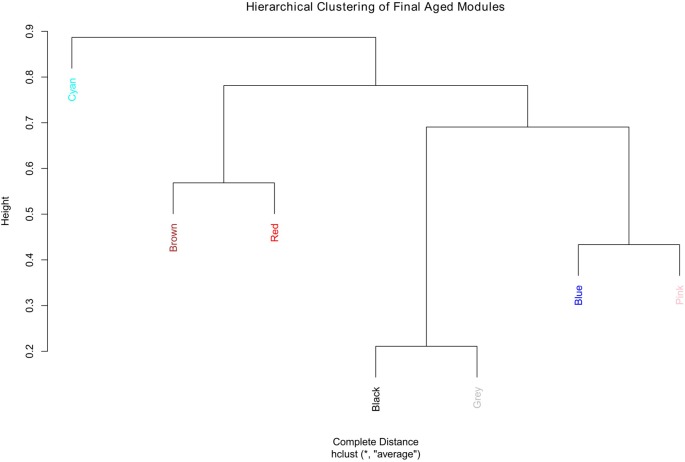
Hierarchical clustering of the final aged modules.

The aged network resulted in many modules, most with small numbers of genes, for example, 13 of the modules had fewer than 300 genes each and nine of them had less than 200 genes each (result not shown). For better comparison, the number of modules in the aged network was brought closer to that of the young network. This was accomplished by merging the modules (Supplementary Figure S10). In order to keep the module numbers similar to that of the young network, a cut height of 0.4 was chosen that generated seven modules in the aged network (including the gray module; Figure 3).

Since module names/labels in a network were randomly generated, the seven aged modules were matched to the seven young modules to check for similarity and module overlap of gene members (Supplementary Tables S3, S4). Once a significant match was found, modules in the aged network were renamed after the matched young network module names. Table 2 shows the final modules in the young and aged networks along with the number of genes belonging to each module. In addition, Table 2 shows which aged modules are matched to which young modules. The black module from the aged network had genes matching significantly to both the blue and brown modules in the young network. The aged brown, red and cyan modules matched to the green, red and yellow young modules, respectively, while the blue and pink aged modules matched a single turquoise young module. This module matching process is helpful when comparing similar modules between networks, for example, aged vs. young.

**Table 2:** Modules in the R7 young and aged networks.

Samples	Module names						
**Young**	**Blue**	**Brown**	**Green**	**Gray**	**Red**	**Turquoise**	**Yellow**
# of genes	1015	759	380	1319	341	1129	731
Aged (original labels**)	**Blue (Black)**	**Brown (Black)**	**Green (Brown)**	**Gray (Gray)**	**Red (Red)**	**Turquoise (Blue and Pink)**	**Yellow (Cyan)**
# of genes	1151	1151	554	2600	206	508 and 366	289

There were seven modules in each group including the gray module. Aged modules were matched to the young modules to find modules containing the maximum number of matching genes. Once identified, the aged module names were changed to match the respective young module names for easy comparison. **Original labels/names of the aged modules before matching to the young modules are in bracket.

**Figure 4:**
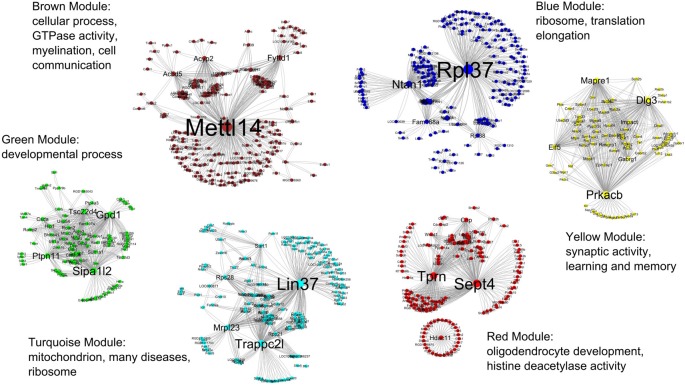
All six modules in the R7 young networks. The modules are represented by the color of their respective names, for example, the blue module is represented by the color blue. The most significant GO functional categories represented by the genes belonging to each module are also shown.

For clarity, only the top 500–600 most connected genes and their co-expression interactions were used to create each module network. Co-expression information from all modules were combined and imported into the Cytoscape for visualization. Figure 4 shows all six modules in the R7 young networks where the modules are represented by the color of their respective names (e.g., the blue module is represented by the color blue).

### Exploring the Functional Significance of Modules

Biological significance analysis of the network modules was performed using the functional annotation clustering analysis in DAVID that utilizes the GO and other biological pathway information databases. DAVID functional annotation clustering analysis was used through the RDAVIDWebService tool in R to identify the most relevant (overrepresented) biological terms associated with each module gene list. The DAVID database offers extended annotation coverage with over 40 annotation categories, including GO terms, protein-protein interactions, protein functional domains, disease associations, bio-pathways, sequence features, homology and many more (Huang et al., 2009b). However, for reasons of simplicity and to better understand the biological significance of the network modules identified above, only the biological processes (BP), molecular functions (MF), and cellular components (CC) GO terms and all KEGG Pathway terms were included in the functional annotation clustering analysis.

Affymetrix probe set identifiers of all the genes belonging to a network module (Table 2) were used as the input gene list. The total number of genes from the RAE230A array for the R7 dataset (after preprocessing and filtering) was 5674, and was used as a background population. *Rattus norvegicus* was used as species. The function getClusterReportFile(…) in RDAVIDWebService was used with default parameters to retrieve all relevant information. Next getClusterReport(. . .) function was used to extract the functional annotation chart file, which was saved as a text file and later analyzed. An enrichment score cutoff of 1.0 was used to minimize the number of clusters that were returned.

Table 3 shows the summary result of GO analysis for the young modules. The most significant GO functional categories represented by the genes belonging to each module are also shown in Figure 3. The results show that, in general, each module is highly enriched with genes functioning in broad but distinct GO functional categories or biological pathways with highly significant enrichment scores.

**Table 3:** GO functional analysis summary for the R7 young modules.

Module	Major GO Categories	*p*-value
Blue	Ribosome, translation elongation	9.85E-08–2.02E-09
Brown	Cellular process, GTPase activity, myelination, cell communication	0.02–0.006
Green	Developmental process	9.36E-04
Red	Oligodendrocyte development, histine deacetylase activity	0.01–0.005
Turquoise	Mitochondrion, many diseases, ribosome	1.20E-04–3.12E-06
Yellow	Synaptic activity, synaptic transmission, learning and memory	2.94E-04–4.77E-15

### Validating Network Modules

Network modules were validated by assessing their preservation and overlap across datasets. This was done by comparing the modules’ gene expression data as follows: R7 young vs. R7 aged; R7 young vs. B8 and K9 young; R7 aged vs. B8 and B7 aged.

#### Module Preservation

Module preservation was assessed quantitatively where the R7 5674 top most connected genes from the young networks were compared to the same genes in other datasets to see how well the module assignment of these R7 genes and their module-wise functions are preserved in other datasets. However, in each comparison the R7-Y network module definition was used as a reference and networks were created from gene expression data accordingly for comparison. For example, in the comparison between R7-Y vs. B8-Y, the same R7 top most connected 5674 genes were selected from B8-Y. Next, the same R7-Y gene module definition was mapped to the B8-Y genes. There was an exception for the R7-A vs. B7-A comparison where only 2140 genes were used because only these genes were common between the two different chip types used in the two independent studies.

**Figure 5:**
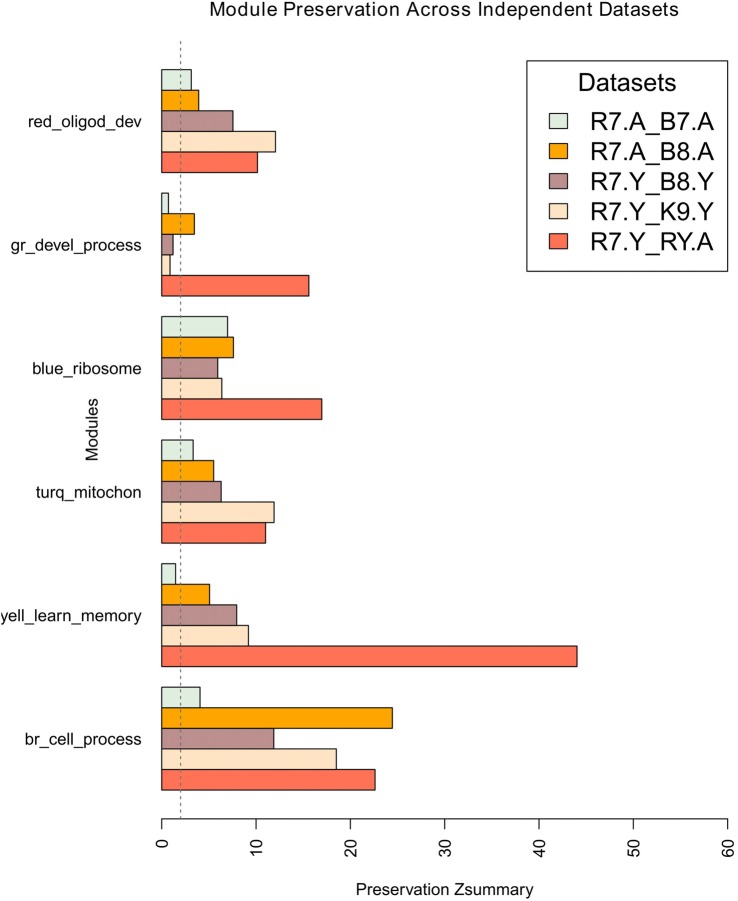
Preservation of R7 young network modules across studies, age and platform. The *x*-axis presents the preservation Z_summary_ statistics and the y-axis represents the R7-Y modules such as brown, yellow, turquoise, blue, green and red along with their major significant functional categories. In each comparison R7 module assignment was used as a reference. The preservation of modules in R7-Y vs. R7-A is shown as a guide. The vertical dotted line at Z_summary_ score 2.0 indicates the borderline between no preservation and very weak preservation. Generally, 5 < *Z* < 10 indicates moderate preservation and Z > 10 indicates high preservation. Legends: gr, green; turq, turquoise; yell, yellow; br, brown.

Module network preservations were estimated by keeping the maximum module size at 700 and using 30 permutations. The results are summarized in the bar plot in Figure 5. It presents the preservation of R7 young and aged modules in each comparison as Z_summary_ statistics along the *x*-axis. All the R7 young modules (e.g., brown, yellow, turquoise, blue, green and red) along with their major significant functional categories are represented in the *y*-axis. Except the green module, all other modules generally show moderate to high preservation across independent studies. The brown module shows the highest preservation among all the modules while the green module shows the lowest preservation. All modules in general in the R7 aged vs. B7 aged comparison shows comparatively lower preservation than in the other comparisons.

#### Module Overlap between Networks

Comparing networks by calculating module overlaps between networks provides another way to validate network modules using independent datasets. We performed a pair-wise comparison for all datasets. After merging datasets by matching genes, there were 3626 top most connectivity genes common between R7 and B8, 3138 between R7 and K9, and 2140 between R7 and B7 networks (Supplementary Table S2).

**Figure 6:**
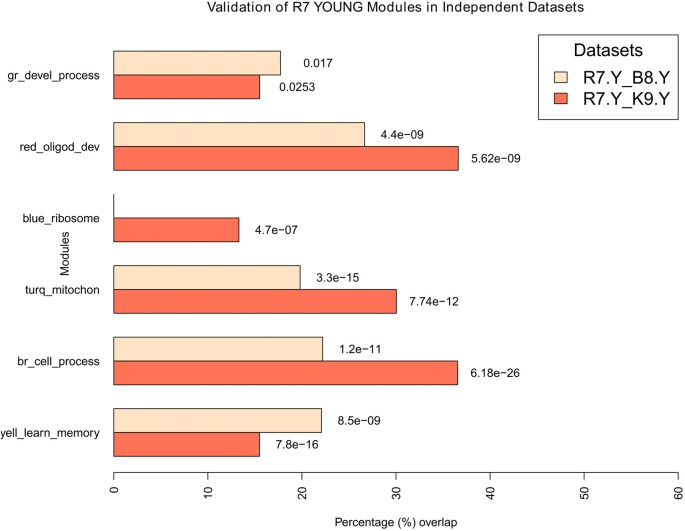
Validation of young modules in independent datasets. All modules in R7-Y were compared for their significant overlaps in B8-Y and K9-Y. The percentage overlap is shown on the *x*-axis and the modules, along with their broad significant GO categories, are shown on the y-axis. Legends: gr, green; turq, turquoise; yell, yellow; br, brown.

**Figure 7:**
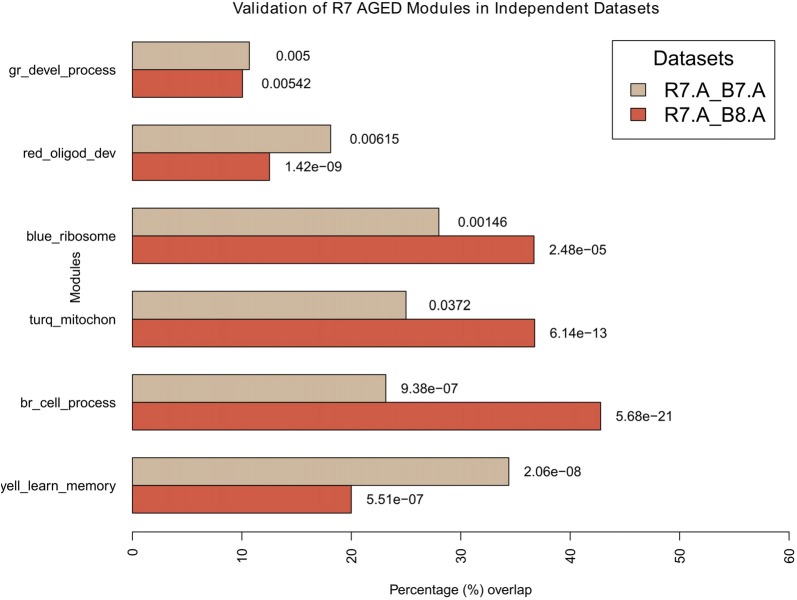
Validation of aged modules in independent datasets. All modules in R7-A were compared for their significant overlaps in B8-A and B7-A. The percentage overlap is shown on the *x*-axis and the modules, along with their broad significant GO categories, are shown on the *y*-axis. Legends: gr, green; turq, turquoise; yell, yellow; br, brown.

Once two datasets had the same matching genes selected, next, for each comparison (e.g., between R7-Y and B8-Y) all modules were compared between the two datasets (i.e., the module assignment of the genes in R7 were matched to the same genes in B8). For each comparison, the results generated an overlap table and a *p*-value table showing the number of genes that matched between each pair of modules and their associated *p*-value significance, respectively. From these results, percentage overlap for each module was calculated by dividing the total genes matched to a module (e.g., number of genes from an R7 module matching to the genes from a module in the second dataset) with the total matched to all modules (e.g., number of genes from an R7 module matching to the genes in all modules (max. shared) in the second dataset). In cases where an R7 module was matched to multiple modules in the second network, overlap with the lowest *p-value* was considered. For example, the R7-Y yellow module genes (731) matched to only 85 genes in the B8-Y red module with the lowest *p*-value (highest match), while they matched to 385 genes in the B8 young network shared by all the modules. Therefore, the percentage overlap is 85/385 = 22.08% with a *p*-value of 8.50e-09. The final results for all four comparisons (column five in Supplementary Table S2) are summarized in the bar plots in Figure 6 for young and in Figure 7 for aged networks.

For the young, all modules in R7-Y were compared for their significant overlap in B8-Y and K9-Y (Figure 6). The results show that except the blue module in the R7-Y vs. B8-Y comparison, all modules show a significant repeatability with a *p*-value < 0.05. The red module showed the maximum overlap trailed by brown, turquoise, yellow, green and blue.

For the aged, all modules in R7-A (using the R7 young module definition) were compared for their significant overlap in B8-A and B7-A (Figure 7). The results show that all modules demonstrate a significant repeatability with a *p*-value < 0.05 across independent datasets. The blue module showed the maximum overlap trailed by turquoise, brown, yellow, red and green.

### Differential Network Analysis of Young vs. Aged

In order to assess the changes in co-expression patterns of the young as they age and how the aging would affect learning impairments, we compared several interesting network modules between young (learning unimpaired) and aged (predominantly learning impaired) networks generated from the R7 data. This comparative investigation involved visualizing them side by side, comparing expression patterns between networks, and searching for key genes. In addition, it involved identifying the key genes’ functions and pathways that can help explain the learning differences as well as the aging effect that had been observed between the young and aged animals. Differential expression levels for the top 5674 genes in the R7 data were calculated by using the *limma* package in Bioconductor. The log fold changes of expression differences between young and aged for all genes were saved as a tab delimited text file, and later loaded as node attributes in Cytoscape for each module.

**Figure 8:**
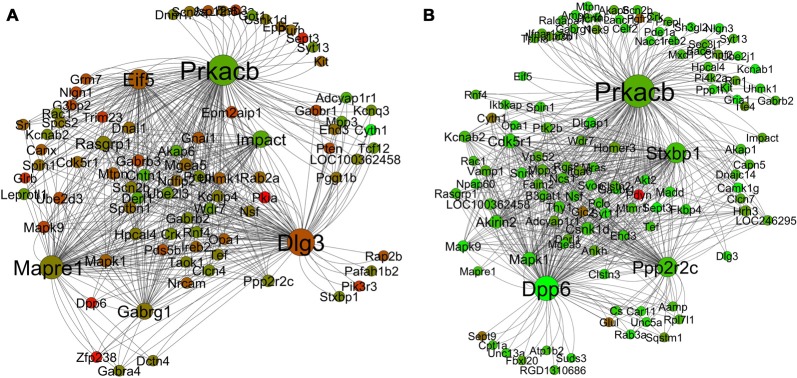
Differential co-expression network analysis of the yellow “learning and memory” module in the young** (A)** and aged **(B)** in R7. The color of each node displays differential expression level (log fold change value) between young and aged samples. Each node size is proportional to the number of co-expression interaction the node has. Legends: red is upregulation; green is downregulation.

The module that is most relevant to this article is the yellow (“learning and memory”) module. Figures 8A,B presents the differential co-expression networks of this module between young (A) and aged (B) rats, which demonstrates a clear difference in expression patterns between the young and the aged genes. The majority of the genes in the aged yellow network show lower expression compared to the young. In addition, the comparative analysis demonstrates differential co-expression for many genes between the two networks (i.e., some genes display more co-expression interaction than others and this varies between the young and the aged networks). The results allow one to identify a number of key ASLI genes for further investigation (see below).

### Identifying and Validating ASLI Candidate Hub Genes

In a co-expression network, genes that are highly connected with many other genes are called hub genes. These genes show significant correlation with the module eigengenes and have high within-module connectivity. After closely studying the networks in young (learning unimpaired) and aged (predominantly learning impaired), we have identified a set of key hub genes in each module. Some of the hub genes in the yellow module in R7 are shown in Table 4. Some of them are already known as learning genes and were identified in our previous meta-analysis (Uddin and Singh, 2013). Table 5 shows the number of significant AY (aged vs. young) meta-analysis genes that are also members of different modules in the R7-Y network. Particularly, it shows that there are 165 AY significant meta-analysis genes in the yellow module. Effect size estimates from the meta-analysis for the above ASLI candidate hub genes are summarized in Supplementary Table S5. In addition, we have created individual forest plots for some of these hub genes, which are presented in Supplementary Figures S11–S25.

**Table 4:** Top candidate age-associated spatial learning impairment (ASLI) hub genes in the yellow module of the R7 dataset.

Hub gene	Function description	Reference
*Camk1g*	Encodes a protein similar to calcium/calmodulin-dependent protein kinase (CaMK), but its exact function is not known. CaMKs activated by the neuronal Ca^2+^ influx phosphorylate cyclic adenosine monophosphate (cAMP) responsive element binding protein (CREB), which has been implicated in spatial learning and memory formation.	Thomas and Huganir (2004) and Voglis and Tavernarakis (2006)
Cdk5r1*	Involved in the pathology of Alzheimer’s disease through the deregulated activity of cyclin-dependent kinase 5 (Cdk5), and also involved in synaptic plasticity, and learning and memory.	Angelo et al. (2006) and Shukla et al. (2012)
*Cntn1*	Contributes to the formation and function of neuronal connections, axon-glia communication, and necessary for myelin sheath formation by oligodendrocytes.	Ranscht (1988) and Çolakoğlu et al. (2014)
Dlg3*	Encodes a member of the membrane-associated guanylate kinase protein family; may play a role in clustering of N-methyl-D-aspartate (NMDA) receptors at excitatory synapses. It is highly enriched in the postsynaptic density (PSD), and plays essential roles in synaptic organization and plasticity.	Elias and Nicoll (2007), Elias et al. (2008) and Wei et al. (2015)
*Dpp6*	Encodes an auxiliary subunit of voltage-gated potassium-4 channels and regulates the A-type K+ current gradient, which regulates dendritic excitability.	Nadal et al. (2003) and Wolf et al. (2014)
*Eif5*	Make 80S ribosomal initiation complex functional for translation.	Si et al. (1996)
*Gabrg1*	Belongs to the ligand-gated ionic channel family. It is an integral membrane protein and plays an important role in inhibiting neurotransmission.	Pirker et al. (2000) and Ye and Carew (2010)
Kcnab2*	Encodes one of the beta subunits of the shaker-related Kv channels (Kv1.1 to Kv1.8) and found as a component of almost all potassium channel complexes containing Kv1 α subunits. It is a learning gene that is known to contribute to certain types of learning	Voglis and Tavernarakis (2006) and McKeown et al. (2008)
Mapk1*	Encodes a member of the MAP kinase family and is known as a learning gene. Hippocampal expression of Mapk1 is essential for synaptic plasticity and spatial learning.	Selcher et al. (2001), Sweatt (2001) and Thomas and Huganir (2004)
*Mapre1*	It is involved in the regulation of microtubule structures and chromosome stability.	Tirnauer et al. (2002) and Kim et al. (2013)
*Ndfip2*	Affects receptor tyrosine kinase signaling by ubiquitinating several key components of the signaling pathways through binding to E3 ubiquitin ligases.	Cristillo et al. (2003) and Mund and Pelham (2010)
*Ppp2r2c*	Ppp2r2c gene encodes one of the four B regulatory subunits of the protein phosphatase 2A (PP2A) enzyme complex. Inhibition of PP2A by inhibitor I1PP2A results in deficits in spatial reference memory and memory consolidation in adult rats.	Xu et al. (2006) and Backx et al. (2010)
*Prkacb*	Encodes the catalytic beta subunit of protein kinase A (PKA). PKA activates CREB and contributes to learning induced gene expression. Prkacb expression is required for LTP in the Hippocampus.	Qi et al. (1996), Howe et al. (2002) and Nguyen and Woo (2003)
Pten*	It modulates activation of the phosphatidylinositol 3-kinase (PI3K)/protein kinase B (Akt) pathway. PTEN independently controls the structural and functional properties of hippocampal synapses and plays a direct role in activity-dependent hippocampal synaptic plasticity such as LTP and LTD.	Maehama and Dixon (1998), Blair and Harvey (2012) and Sperow et al. (2012)
*Rasgrp1*	It is a guanine nucleotide-exchange factor. When it is activated by Ca^2+^/calmodulin and diacylglycerol (DAG), it facilitates exchange of GDP to GTP and activates Ras.	Stone (2006)
*Scn2b*	Scn2b is a complex glycoprotein comprised of an alpha subunit and often one to several beta subunits. It was reported to have a role in epilepsy.	Baum et al. (2014) and XiYang et al. (2016)
*Stxbp1*	Plays a role in release of neurotransmitters via regulation of syntaxin, a transmembrane attachment protein receptor.	Kurps and de Wit (2012)

Genes with an “*” were also identified as learning genes in a previous meta-analysis (Uddin and Singh, 2013). Legend: *Camk1g*, Calcium/calmodulin-dependent protein kinase I gamma; *Cdk5r1*, Cyclin-dependent kinase 5, regul. subunit 1 (p35); *Cntn1*, Contactin 1; *Dlg3*, Discs, large homolog 3; *Dlgap1*, Discs, large homolog-associated protein 1; *Dpp6*, Dipeptidyl-peptidase 6; *Eif5*, Eukaryotic translation initiation factor 5; *Gabrg1*, Gamma-aminobutyric acid (GABA) A receptor, gamma 1; *Impact*, Impact RWD domain protein (RWDD5); *Kcnab2*, Potassium channel, voltage gated shaker related subfamily A regulatory beta subunit 2; *Mapk1*, Mitogen-activated protein kinase 1 (ERK); *Mapre1*, Microtubule-associated protein, RP/EB family, member 1; *Ndfip2*, Nedd4 family interacting protein 2; *Ppp2r2c*, Protein phosphatase 2, regulatory subunit B, gamma; *Prkacb*, Protein kinase, cAMP-dependent, catalytic, beta; *Pten*, Phosphatase and tensin homolog; *Rasgrp1*, RAS guanyl releasing protein 1 (calcium and DAG-regulated); *Scn2b*, Sodium channel, voltage-gated, type II, beta; *Stxbp1*, Syntaxin binding protein 1.

**Table 5:** Significant AY meta-analysis genes (Uddin and Singh, 2013) common in R7-Y modules.

R7 Modules	Number of genes	Number of AY meta-analysis genes matching to each module
Blue	1015	275
Brown	759	195
Green	380	130
Gray	1319	334
Red	341	133
Turquoise	1129	275
Yellow	731	165

The candidate ASLI hub genes were checked for their repeatability in networks constructed independently from B8, K9, and B7. The results are summarized in Table 6. Details of the hub gene validation data are available in Supplementary Tables S6–S11. The results show that a number of hub genes from the yellow module are repeated in one or more independent datasets in B8, K9 or B7 with a *p*-value ≤ 0.05. From the R7 yellow module *Prkacb*, *Scn2b*, *Cntn1*, *Pten* and *Ndfip2* were found present as hub genes in the K9 network; *Dlgap1* was found in the B7 and B8 networks; and *Camk1g* was found repeated in the B7 network. Notably, many of these hub genes were in the list of top 20 mean KME values in other networks, but their *p*-values were not significant, for example, *Dlg3*, *Mapre1*, *Dpp6*, *Stxbp1*, *Impact* and *Mapk1*.

**Table 6:** Significant ASLI candidate hub genes from the yellow “learning and memory” module and their repeatability in independent datasets.

Gene symbol	Number of co-expression in R7 network		Hub gene repeated in study	*t*-test	Known learning gene
	Young	Aged		*p*-value	
*Camk1g*	0	4	B7-A	0.0003	No
*Cdk5r1*	5	22			Yes
*Cntn1*	6	0	K9-Y	0.0186	No
*Dlg3*	63	1			Yes
*Dlgap1*	0	7	B7-A, B8-A	0.0332	No
*Dpp6*	2	68			No
*Eif5*	36	1			No
*Gabrg1*	23	1			No
*Impact*	24	1			No
*Kcnab2*	2	10			Yes
*Mapk1*	9	19			Yes
*Mapre1*	49	1			No
*Ndfip2*	4	0	K9-Y	0.0217	No
*Ppp2r2c*	6	47			No
*Prkacb*	76	103	K9-Y	0.0523	No
*Pten*	2	0	K9-Y	0.0308	Yes
*Rasgrp1*	15	5			No
*Scn2b*	5	1	K9-Y	0.0028	No
*Stxbp1*	1	49			No

## Discussion

In this research, we explored the idea that recent mathematical modeling approaches have the potential to fully utilize the gene interaction information present in microarray data and to help identify useful new candidate genes and their networks. In this respect, we investigated the use of co-expression networks using WGCNA for the first time in the analysis of ASLI microarray gene expression data. The data represented young rats that were learning unimpaired and aged rats that were predominantly learning impaired. This allowed us to identify a set of network modules and ASLI candidate hub genes. These modules and candidate hub genes are repeatable across independent datasets. The implications of major findings are discussed below.

### Co-Expression to Co-Functionality—From the Perspective of Modules

One useful property of a co-expression network is module. In a module the expression patterns of the genes are mutually correlated (Langfelder and Horvath, 2008). The focus on co-expression modules, each consisting of possibly hundreds of genes with common co-expression across samples, allows for a biologically motivated reduction of data while also alleviating the problem of multiple comparisons (Levine et al., 2013). Further, just as correlated genes tend to have similar biological functions, on a larger scale, modules tend to contain genes with similar biological functions (Lee et al., 2004).The results obtained in this research and the follow up network analysis support these hypotheses. For example, the use of WGCNA reduced R7 data into a few biologically meaningful co-expression modules. The follow up GO analysis and literature search results were persuasive enough to indicate that each module gene set likely serve a distinct major biological function, thus, pointing to the widely held notion of “co-expression to co-functionality”. It is important to note that the networks and modules constructed from R7 microarray data were based on the gene expression patterns alone (i.e., there was no prior knowledge of the genes’ function at the time of network construction). Once the networks were divided into modules and their module-wise GO functional analysis was performed, it was indeed observed that each module pointed to a broad but distinct category of biological function, and genes in each module shared similar subcategories of functions all converging to the broad functional category of the module (Table 3, Figure 4). Particularly, the genes in the yellow module showed significant enrichment in GO functions and pathways related to learning and memory formation in the brain. This is expected as the young and aged rats used in the original research were tested for their memory performances where the experimental young animals displayed clear learning unimpairment and the majority of the aged animals demonstrated learning impairment. The inclusion of various controls, including the aged animals that demonstrated learning unimpairment, served as controls e.g., for stress, physical ability as well as for factors that may contribute to learning irrespective of age. It is likely that the learning unimpaired aged rats might also display sign of learning impairment as they progress towards further aging. Although, the other modules are enriched with functions not directly related to learning and memory, they are critical for normal neuronal processes such as communication, growth, development and maintenance. For example, genes in the brown module are significantly enriched in functions contributing to the various cellular processes and communication, the green module genes in developmental processes, and the red module genes in oligodendrocyte development.

Thus, alteration of these modules’ normal module-wise functions at old age through altered gene expression, as observed in the datasets, has the potential to affect normal functioning of learning and memory formation process. Preservation of these modules were not only validated across networks created from independent datasets, but also the gene members of these modules demonstrated significant module membership (module overlap) across the independent networks (Figures 6–8).

Gene co-expression analysis studies in multiples species, tissues and platforms have shown that co-expressed genes tend to be functionally related (Williams and Bowles, 2004; Obayashi et al., 2008; Oldham et al., 2008). In order to investigate, whether observed clusters or modules of co-expressed genes are of functional significance, Lee and Sonnhammer (2003) observed that genes involved in the same biochemical pathways tend to be clustered together in a number of eukaryotic genomes. By a heuristic generalization known as “guilt by association”, it has been computationally established that functionally related genes are organized into co-expression networks, in practice assisting functional annotation of uncharacterized genes (Michalak, 2008). For example, physically interacting proteins in yeast were found to be encoded by co-expressed genes (Ge et al., 2001; Wuchty et al., 2006). These observations likely have inspired the development of co-expression network analysis methods. Gene network modeling using co-expression approaches provide insight into cellular activity as genes that are co-expressed often share common functions (Piro et al., 2011). Such networks have been widely used to study many diseases and phenotypes because of their ease of use and their ability to provide more biologically meaningful results (Gargalovic et al., 2006; Chen et al., 2008; Min et al., 2012; Zhou et al., 2014; Holtman et al., 2015; Maschietto et al., 2015; Rickabaugh et al., 2015; Spiers et al., 2015; Ye and Liu, 2015).

Microarray data captures functional relationship among genes that can provide biologically relevant information. In traditional microarray data analysis, however, these relationships remain essentially unexplored. Thus, a modular approach to gene function through WGCNA provides a sensible way to extract such functional information from large microarray datasets in a biologically meaningful way. Particularly, this analysis has shown that specific learning associated functional gene modules can be identified through co-expression network modeling where genes in the module show significant enrichment in learning and synaptic plasticity related GO functions.

### Gene Co-Expression to Co-Functionality—From the Perspective of Hub Genes: New Insight into the Molecular Mechanisms of Learning and Memory Formation

Hub genes play a central role in the structure of co-expression networks as they are often relevant to the function of regulatory networks. The ability to efficiently transit cellular signals within and between co-expressed clusters is facilitated by “hubs”, which are connected to a large number of nodes (Gaiteri et al., 2014). Analysis of the yeast protein-protein interaction network revealed that highly connected nodes are more likely to be essential for survival (Jeong et al., 2000; Carter et al., 2004; Han et al., 2004). The co-expression networks of the yellow “learning and memory” module (Figure 8) display a tight interrelationship of a large number of nodes with some hub genes. What is most interesting is that the co-expression of these hubs and nodes, as demonstrated in this network analysis, is not a random aggregation of some genes. Literature review suggests that the correlated expression pattern of the hub genes in the yellow networks (Figure 8A) may in fact be highly coordinated, and inside the young rats’ hippocampus they may be serving a common purpose. The combined effect of the functions of the hub genes that are co-expressing together in individual modules may in fact contribute to the co-functionality of the whole module. The purpose could be to maintain the functional integrity of the normal process of learning and memory formation mechanisms, which are disrupted in the aging brain. Indeed, the side-by-side comparison (Figure 8) of the yellow module networks between young and aged rats demonstrates a clear difference in expression patterns. The majority of the genes in the aged yellow network show lower expression compared to the young. In addition, the comparative analysis demonstrates differential co-expression for many genes between the two networks. These genes display more co-expression interaction than others and the number of interaction varies between the young and the aged networks. For example, the gene *Dlg3* has 63 co-expression connections with other genes in the young network while only one in the aged. Similarly, *Dpp6* has only two co-expression connections with other genes in the young network while 68 in the aged (Table 6). Since, such genes with many co-expression connections with other genes in a network often play a role as hubs; we have short listed 19 of these genes as candidate ASLI hub genes from both the young and aged yellow module networks. These genes include *Camk1g*, *Cdk5r1*, *Cntn1*, *Dlg3*, *Dlgap1*, *Dpp6*, *Eif5*, *Gabrg1*, *Impact*, *Kcnab2*, *Mapk1*, *Mapre1*, *Ndfip2*, *Ppp2r2c*, *Prkacb*, *Pten*, *Rasgrp1*, *Scn2b* and *Stxbp1* (Table 4). The results show that some of these hub genes are already known as key learning and memory genes and have well established roles in memory functions. While for others, information is emerging indicating their direct or indirect role in learning and memory. Below we summarize what is already known from the literature about the molecular mechanisms of learning and memory formation and how the candidate ASLI hub genes from this research fit into that big picture.

#### Role of *Camk1g*, *Dlg3*, *Dlgap1*, *Dpp6*, *Kcnab2*, *Mapk1* and *Stxbp1* in CREB Related Pathways

Several major signaling pathways seem to modulate synaptic plasticity mechanisms in the brain and have been implicated in learning and memory formation processes (Sweatt, 2001; Nguyen and Woo, 2003; Ye and Carew, 2010; Baudry et al., 2015). Some of the major pathways relevant to this study include the PKA, CaMKs, MAPK and PI3K/Akt pathways that have been implicated in LTP formation. LTP is a synaptic plasticity mechanism and a cellular correlates thought to underlie learning and memory. Following external stimulation, a set of crucial upstream events are necessary for their activation, which include NMDA receptors and the resulting calcium influx.

Calcium-dependent phosphorylation of CREB is primarily caused by PKA, CaMK and MAP kinase, which leads to prolonged CREB phosphorylation. CREB in turn contributes to the transcription of a set of immediate early genes implicated in learning and memory formation. CREB is thought to mediate long-lasting changes in brain function. For example, CREB has been implicated in spatial learning, behavioral sensitization, long-term memory of odorant-conditioned behavior, and long-term synaptic plasticity (Thomas and Huganir, 2004; Alberini, 2009; Chen et al., 2010; Sweatt, 2010). The ASLI candidate hub genes that are important in the CREB related pathways include *Camk1g*, *Dlg3*, *Dlgap1*, *Dpp6*, *Kcnab2*, *Mapk1* and *Stxbp1*. For example, *Stxbp1* plays a role in releasing of neurotransmitters via regulation of syntaxin (Kurps and de Wit, 2012) and may serve to transfer of signal through the synapse. *Dlg3*, also known as synapse-associated protein 102 (SAP102), is a scaffolding protein highly enriched in the postsynaptic density (PSD), and plays an essential role in synaptic organization and plasticity (Elias and Nicoll, 2007). *Dlg3* interacts directly or indirectly with major types of glutamate receptors. It binds directly to *N*-methyl-d-aspartate receptors (NMDARs), anchors receptors at synapses, and facilitates transduction of NMDAR signals (Wei et al., 2015).

CaMKs, particularly CaMKII has been shown to be directly activated by calcium influx through the NMDA receptor. CaMKs play a significant role in learning and memory formation through the activation of CREB signaling (Sweatt, 2001; Bito and Takemoto-Kimura, 2003; Thomas and Huganir, 2004; Baudry et al., 2015). It is very likely that *Camk1g*, which has not been reported before in relation to memory impairment, may function in a similar manner. It is likely that down-regulation of *Camk1g* in the aged rats may in fact contribute to ASLI in those animals through the CaMK pathway to modulate CREB phosphorylation.

*Camk1g* co-expression with other learning genes such as *Mapk1*, *Kcnab2* and *Dpp6*, functioning in the MAPK pathway or in various ion channels indicate a potential co-functioning of these genes towards learning and memory formation. Some may involve a feed-back loop type activation/mechanism. For example, during the early phase of LTP at postsynaptic terminals of CA1 hippocampal neurons, calcium entering through α-amino-3-hydroxy-5-methyl-4-isoxazolepropionic acid (AMPA) and NMDA receptors activates CaMKII, which phosphorylates Kv channels and increases neuronal excitability (Sweatt, 2001). Similarly, *Mapk1*, stimulated by elevated levels of cAMP as a result of calcium entry and subsequent activation of adenylyl cyclase-1, phosphorylates the A-type potassium channel (Kv1.4 and Kv4.2) resulting in increased depolarization, allowing influx of Ca^2+^ through the NMDA and voltage-gated Ca^2+^ channels, which results in increased cAMP levels in the hippocampus in mice. The increase in Ca^2+^ and cAMP induces the MAPK pathway. Thus, the induced pathway activates additional pools of MAPK1, some of which can further increase phosphorylation of Kv1.4 and Kv4.2, whereas others may phosphorylate nuclear targets. Voltage-gated potassium (Kv) channels play important roles in regulating the excitability of neurons and other excitable cells. Subthreshold activating, rapidly inactivating, A-type K^+^ currents are non-uniformly expressed in the primary apical dendrites of rat hippocampal CA1 pyramidal neurons, with density increasing with distance from the soma (Hoffman et al., 1997). These changes correlate with impaired spatial memory and context discrimination (Morozov et al., 2003). Note that the ASLI candidate gene *Kcnab2* encodes one of the beta subunits of the Kv channels (Kv1.1 to Kv1.8) and this subunit is found as a component of almost all potassium channel complexes containing Kv1α subunits (McKeown et al., 2008). Deletion of *Kcnab2* in mice leads to deficits in associative learning and memory and loss of this gene function likely contributes to the cognitive and neurological impairments in humans (Voglis and Tavernarakis, 2006; Perkowski and Murphy, 2011). The role of *Mapk1* through MAPK (ERK) signaling is not only documented in LTP, but also in spatial learning (Blum et al., 1999; Selcher et al., 2001; Sweatt, 2001; Thomas and Huganir, 2004). DPP6 may take part by regulating the A-type K+ current gradient, ultimately contributing to synaptic integration and dendritic excitability (Nadal et al., 2003; Wolf et al., 2014). The action potential firing and dendritic excitability must be balanced by inhibition in hippocampal neuron. This is likely achieved by *Gabrg1* (Costa et al., 2002; Cui et al., 2008) and a number of other GABA receptors that demonstrated co-expression in the yellow module.

Dendritic integration of synaptic inputs is fundamental to information processing in neurons of diverse function, serving as a link between synaptic molecular pathways and higher-order network function (Sun et al., 2011). Dendritic ion channels play a critical role in regulating such information processing and are targets for modulation during synaptic plasticity (Shah et al., 2010). Normal experience-dependent changes in the excitability of dendrites (dendritic plasticity), involving the down-regulation of A-type K^+^ currents by down-regulation of *Dpp6* (observed here), may represent a mechanism by which neurons store recent experience in individual dendritic branches (Makara et al., 2009). Down-regulation of *Kcnab2* may contribute to the reduction of A-type potassium channel currents through reduced availability of Kv1.4. Future studies are required to investigate the effect of *Dpp6* and *Kcnab2* in synaptic development and spatial memory formation.

#### Role of *Prkacb* in the PKA Pathway

*Prkacb*, a new ASLI candidate in the PKA pathway, once activated by a variety of upstream signals, including calcium, can phosphorylate and regulate a variety of downstream signaling cascades linked to regulation of transcription and translation (Baudry et al., 2015). It can phosphorylate AMPA and NMDA receptors and regulate their functions. PKA plays a major role in long-term changes in synaptic strength in the brain (Nguyen and Woo, 2003) and has been well known for its critical role in learning and memory formation (Waltereit and Weller, 2003). There are direct genetic evidence that the *Prkacb* isoform is required for long-term depression, long-term potentiation and depotentiation in the hippocampus (Qi et al., 1996; Howe et al., 2002).

#### Role of *Ndfip2*, *Pten* and *Rasgrp1* in the PI3K/Akt and Related Pathway

Another pathway that is making itself relevant in this big picture is the PI3K/Akt pathway. A set of genes involved here include the ASLI candidate genes *Ndfip2*, *Pten* and *Rasgrp1*. *Ndfip2* and *Pten* were down-regulated in the aged compared to the young (effect size = −0.38, *p*-value = 0.22 and effect size = −0.37, *p-value* of 0.01, respectively). In the brain, tyrosine kinase receptor TrkA is phosphorylated on the plasma membrane by the binding of another growth factor NGF, which later activates three major signaling pathways: the PI 3 kinase pathway leading to activation of Akt kinase, the ras pathway leading to MAP kinases, and the PLC pathway leading to release of intracellular Ca^2+^ and activation of PKC (Purves et al., 2004). *Ndfip2* affect tyrosine kinase signaling pathway through Nedd4 ligases (Cristillo et al., 2003), which associate with EGF receptor and *Pten* (Blair and Harvey, 2012; Sperow et al., 2012). Based on literature information it can be hypothesized that *Ndfip2* may modulate the EGF signaling cascade (Mund and Pelham, 2010); it is possible that *Ndfip2* might be working in the same fashion as NGF in the brain to influence not only Akt kinase pathway through Akt, but also other pathways such ras, MAPK, and PLC. In fact, EGF and NGF share the same Raf → MEK → MAPK pathway to promote distinct outcomes (Vaudry et al., 2002). However, EGF and NGF likely work differently and on different receptor tyrosine kinases (Lee et al., 2002). Therefore, the role of *Ndfip2* in learning and memory can be investigated in a future experiment.

MAPKs are normally inactive in neurons but become activated when they are phosphorylated by other kinases. In fact, MAPKs are part of a kinase cascade in which one protein kinase phosphorylates and activates the next protein kinase in the cascade (Purves et al., 2004). The extracellular signals that trigger these kinase cascades are often extracellular growth factors that bind to receptor tyrosine kinases that, in turn, activate monomeric G-proteins such as Ras. *Rasgrp1*, once activated by Ca^2+^/calmodulin and diacylglycerol (DAG), facilitates the exchange of GDP for GTP and may trigger downstream *Mapk1* signaling (Stone, 2006). Once activated, MAPKs can phosphorylate transcription factors, proteins that regulate gene expression, and may contribute to long-term memory formation (Adams and Sweatt, 2002; Sharma et al., 2003). Indeed, *Rasgrp1* may be a novel link between molecules activated in behavioral paradigms such as phospholipase C and the well-known Ras–MAPK pathway (Buckley and Caldwell, 2004).

Although, *Pten* is known to play a direct role in regulating hippocampal synaptic plasticity, the precise mechanisms underlying *Pten* modulation of synaptic plasticity such as LTP and LTD are not fully known. Recent studies suggest its involvement in postsynaptic mechanism as PTEN inhibition promotes AMPA receptor trafficking to synapses leading to a persistent increase in excitatory synaptic strength in adult hippocampal slices (Moult et al., 2010). On the other hand, enhanced PTEN lipid phosphatase activity has been reported to depress excitatory synaptic transmission, which in turn is required for NMDA receptor-dependent LTD (Jurado et al., 2010). In light of this research, *Pten* is an excellent candidate to study further for it potential involvement in ASLI and the mechanisms in play.

#### Role of *Cntn1*, *Mapre1* and *Ppp2r2c* in Learning and Memory

Co-expression of genes like *Cntn1*, *Mapre1*, etc., which have known functions in neuronal structure, indicates that these genes play an essential role in learning and memory along with other genes discussed above. For example, *Mapre1* is well known to regulate microtubule dynamics (Tirnauer et al., 2002). It plays a crucial role in ADNP function along with other molecules including *Dlg4* and offer protection against cognitive deficiencies in mice (Oz et al., 2014). *Cntn1* is necessary for myelin sheath formation by oligodendrocytes and provides critical signal in axon-glia communication (Ranscht, 1988; Çolakoğlu et al., 2014). *Ppp2r2c*, another new ASLI candidate gene, forms a part of PP2A (protein phosphatase 2A) enzyme complex, which catalyzes a broad range of substrates (Xu et al., 2006). *Ppp2r2c* has been suggested to have a role in synaptic plasticity and hence learning and memory (Backx et al., 2010).

## Summary

Taken together, this research has identified a set of candidate hub genes that all co-express together in a single gene network module. These genes are known to participate in multiple different cellular signaling pathways such as PKA, MapK and CamK as discussed above. Overall, reversible phosphorylation of proteins by kinase and phosphatase enzymes constitutes some major forms of signaling (Backx et al., 2010). These different signaling cascades converge on a common set of mechanisms: (1) post-translational protein modifications; (2) translational regulation; and (3) regulation of gene expression (Purves et al., 2004; Sweatt, 2010; Baudry et al., 2015). Ultimately, these mechanisms are linked to a few of the common events responsible for LTP such as increased number of postsynaptic receptors, and increased dendritic spines. In fact, these mechanisms are not isolated; rather, multiple cross-talk between the signaling pathways exist, which suggests that depending on the conditions, various form of LTP or LTD can be triggered with different features (Middei et al., 2014; Baudry et al., 2015). Thus, the signaling pathways are involved in the mechanism of synaptic plasticity, which in turn is the molecular mechanism for learning and memory (Sweatt, 2001; Barco et al., 2006; Chen et al., 2010). Thus, co-expression of the hub genes along with other genes in the yellow module seems to be leading to a common function in the hippocampus in the brain, which in this case is ASLI. Results from the meta-analysis for these genes strengthen this conclusion (Supplementary Figures S11–S25). The combined meta-analysis results for these hub genes show that they were expressed at a very low level in the brain with comparatively lower standardized mean differences between young and aged, and thus failed to appear towards the top in the differentially expressed aging or learning gene list (Supplementary Tables S1, S2 in Uddin and Singh, 2013). Down-regulation of the majority of the hub genes in the aged rats (Figure 8B) may play a critical role in the spatial learning impairment in the Morris water maze protocol. Interestingly, many of the hub genes’ individual expression patterns follow what is reported in the literature in respect to their potential role in aging associated learning and memory impairment, for example *Camk1g*, *Dlg3*, *Dpp6*, *Mapk1*, *Mapre1*, *Ndfip2*, *Ppp2r2c*, *Pten*, *Prkacb* and *Rasgrp1*. Some other hub genes such as *Cdk5r1*, *Cntn1*, *Impact*, *Kcnab2*, *Scn2b* and *Stxbp1* may have more indirect role. The main function of this second category of genes may involve contributing to the regulation of normal neuronal structure and functions, dysregulation of which become vulnerable at old age, and thus may indirectly contribute to the overall instability of the memory formation mechanism.

In this research, the findings of a specific “learning and memory” module and the associated key hub genes with their known role in learning and memory formation offer a promising insight and a plausible logical expansion to our existing knowledge about the molecular correlates of the mechanisms underlying memory formation, synaptic plasticity and age-associated learning impairment.

### Differential Expression vs. Differential Co-Expression vs. Differential Connectivity

Differential co-expression refers to changes in gene-gene correlations between two sets of phenotypically distinct samples (de la Fuente, 2010). Changes in gene-gene correlation may occur in the absence of differential expression, meaning that a gene may undergo changes in regulatory pattern that would be undetected by traditional differential expression analysis (Gaiteri et al., 2014). The fact that the altered regulatory patterns observed within tissues across phenotypic states in manners that are reflected in altered co-expression networks has been shown in aging mice (Southworth et al., 2009), across corticolimbic regions in major depression (Gaiteri et al., 2010) and between miRNA’s in Alzheimer’s disease (Bhattacharyya and Bandyopadhyay, 2013).

What becomes apparent is that the differential expression and differential co-expression may point to distinct cellular mechanisms involved in ASLI, which may be working in different ways in the cell. For example, our differential expression meta-analysis has identified a large number of genes showing significantly altered expression in the aged rats compared to young rats (Supplementary Tables S1, S2 in Uddin and Singh, 2013). These genes include many immediate early (e.g., *Arc*) or late phase genes (during gene expression) as well as other genes contributing to aging and ASLI. Major functions disrupted by these genes include cell viability, axonogenesis, quantity and synthesis of IP3 and formation of cells.

On the other hand differential co-expression analysis presented here identified a set of modules each with distinct functions. In addition, it has identified a set of candidate ASLI hub genes in one of those modules. From the known function of these hub genes as explained above, it is evident that many of these genes function as kinases and phosphatases in the neuronal information flow process, starting from the synaptic junctions/synapses to the nucleus to activate various transcription factors. Though scattered in different networks, meta-analysis has also identified few hub genes functioning as kinases or in ion channels. Thus the hub genes may be triggering one or more mechanisms that activate other key factors in a number of pathways, which set the stage for the expression of several immediate early or late phase genes, which again most likely activate the expression of majority of the differentially expressed genes. Learning in the young animals most likely induces such mechanisms that synchronously regulate transcription of multiple genes, and may potentially generate co-expression relationships.

Another important observation to note is that all the learning related genes identified in the differential expression and pathway analyses (and genes they generally interact with) are scattered in different networks and pathways (Appendix 6.3.1 to Appendix 6.4.4 in Uddin, 2015). In contrast, the current differential co-expression analysis identified many known learning genes (or genes that appear to be contributing to learning and memory functioning) that are highly concentrated and co-expressed in the yellow “learning and memory” module.

Interestingly, the candidate ASLI hub genes are expressed at a comparatively lower level, with small differences in expression (e.g., effect size) between young and aged samples. For example, the *Prkacb* hub gene was not known to be a learning gene (Supplementary Tables S3, S4 in Uddin and Singh, 2013). Like *Prkacb*, most of the hub genes failed to show significant effect size or differential expression values and remained undetected in the meta-analysis (Supplementary Tables S1, S2 in Uddin and Singh, 2013). This fact highlights the importance of alternate analysis like WGCNA to identify genes that are not detected using the traditional methods. Similar observations have been demonstrated in past studies (Rhinn et al., 2012). For example, the alpha synuclein gene variant “aSynL”, containing a long 3′sUTR, was identified as the most differentially coexpressed gene in several Parkinson’s disease datasets. However, aSynL was not highly differentially expressed and thus would have likely been overlooked by traditional microarray analysis (Gaiteri et al., 2014). Thus, through the identification of modules and hubs, differential co-expression analysis can be used to prioritize specific phenotype-related important molecules.

Another very interesting property of co-expression networks is the network connectivity. Our findings (Supplementary Figure S26) support the newly emerging hypothesis (Oldham et al., 2006; Miller et al., 2008) that differential connectivity is different from differential expression. During the network construction process, I selected genes with high connectivity and filtered out all low connectivity genes (Supplementary Table S2). The observation is that the resulting network modules represent a set of highly connected genes as hubs that were virtually absent in the differentially expressed top gene list (in Uddin and Singh, 2013) and vice versa. In fact, it has been reported that gene-gene correlations in disease can occur with or without changes in expression (Hudson et al., 2009). In addition, differentially expressed genes in some complex psychiatric diseases can have low connectivity, which reside on the periphery of co-expression networks for neuropsychiatric disorders such as depression, schizophrenia and bipolar disorder (Gaiteri and Sibille, 2011; Gaiteri et al., 2014).

## Future Directions

The candidate ASLI genes (including hub genes) and gene networks identified in this research become excellent candidates for further investigations. Particularly, the hub genes can provide a different perspective on gene regulation as they can serve as excellent targets to examine the biological significance of a network module. They could be targeted to see not only a perturbation effect of altered regulation on network module structure and function, but for therapeutic use as well. Co-expression modules are not in fact completely modular as there are often correlations among the members of different modules (Gaiteri et al., 2014). Therefore, any perturbation effect will likely extend outside of a module and will need to be studied. Since, differential co-expression is likely related to altered gene regulation, experiments involving ChIP, or ChIP-seq of potential transcription factors, can be designed to capture related gene regulatory mechanisms after any perturbation. Epigenetic mechanism are also intimately involved during the gene expression process in learning and memory formation (Levenson and Sweatt, 2005, 2006; Gräff and Mansuy, 2008; Franklin and Mansuy, 2010; Sweatt, 2010). So, changes in chromatin structure, methylation and acetylation pattern, as well as miRNA population changes should also be investigated.

For the purpose of future investigation, the candidate ASLI hub genes could be grouped into three categories: (1) Hub genes whose role in learning (including spatial learning) is more transparent than others (i.e., gene with well-established roles in memory, for example, *Camk1g*, *Dlg3*, *Mapk1*, *Ppp2r2c* and *Prkacb*); (2) Hub genes (e.g., *Cdk5r1*, *Cntn1*, *Scn2b*, *Stxbp1*, *Eif5* and *Gabrg1*) where there is not enough information in the literature to support which direction their expression pattern contributes to the ASLI phenotype; and (3) Hub genes where information is emerging indicating their direct or indirect role in learning and memory (e.g., *Pten*, *Kcnab2*, *Mapre1*, *Ndfip1*, *Rasgrp1* and *Dpp6*).

One way to learn the specific effects of hub genes is through knockout experiments. This is because the hub genes are likely to act as drivers of the disease status due to their key positions in the gene networks (Allen et al., 2012). It is known that transmission of signal through scale-free cellular networks is unlikely to be affected by random node deletion; rather it is especially vulnerable to targeted hub attack (Albert et al., 2000). This observation is supported by examples from multiple molecular and brain networks in which hub targeting leads to crucial functional impairment (Stam et al., 2007). Practically, hub genes have been the specific focus for investigations into many disease-correlated modules (Miller et al., 2008; Ray et al., 2008; Torkamani et al., 2010; Voineagu et al., 2011; Maschietto et al., 2015; Ye and Liu, 2015). Analysis of hub genes has been shown to be a promising approach in identifying key genes in many other phenotypic conditions (Kendall et al., 2005; Mani et al., 2008; Slavov and Dawson, 2009; Nibbe et al., 2010; Zhou et al., 2014; Holtman et al., 2015; Rickabaugh et al., 2015; Spiers et al., 2015). Such genes are often of biological interest because of their critical involvement in regulatory pathways or sub-networks and these genes often incur a substantial effect on the pathways as a whole. The candidate ASLI hub genes identified in this research may very likely present a snapshot of what is going on inside brain cells during the memory formation process.

## Conclusion

Despite significant research in the past, ASLI genes and networks remain largely unclear and were the main focus of this article. The major goal of this research was to identify genes and gene networks in ASLI in rats from multiple independent but related gene expression datasets.

In order to overcome some limitations in traditional meta- and pathway analysis, we explored the option of using a mathematical modeling approach that could better utilize the information captured in microarray data. We chose to use WGCNA and applied it on a set of R7 exploratory datasets containing young rats that were learning unimpaired and aged rats that were predominantly learning impaired. This analyses identified a set of gene network modules. To our satisfaction, WGCNA offered a way of prioritizing the molecules solely based on data and without any knowledge of their functions (i.e., by grouping genes into co-expressing network modules). This finding was confirmed by the follow up GO analysis which showed that each module is highly enriched with genes functioning in some broad but distinct GO functional categories or biological pathways. Further, these modules show significant repeatability in independent young and aged validation datasets. Interestingly, this analysis identified a single learning and memory related module and within the module a set of unique hub genes related to ASLI. Majority of the candidate ASLI hub genes from this module remained undetected in our previous meta- and differential expression analysis. Some of these hub genes also show significant repeatability in networks generated from independent validation datasets. These hub genes are highly co-expressed with other genes in the “learning and memory” module. In network comparison between young and aged, these genes not only show differential expression but also differential co-expression and differential connectivity. These likely explain the spatial learning impairment that was observed in the aged rats compared to the young. The known function of these hub genes indicate that they play key roles in critical pathways relating to synaptic plasticity and memory formation. Collectively, they provide a deeper understanding of the mechanisms that may be involved. These candidate ASLI hub genes seem highly promising to investigate further to understand the regulatory networks in ASLI.

Co-expression network analysis as applied in this research shows how to transform large-scale gene expression microarray data involving spatial learning impairment in rats into several testable hypotheses related to ASLI. This type of analysis can complement traditional analysis of microarray data and can help better understand how genes interact with each other, how they are regulated, and what target genes they may affect in order to elucidate the mechanisms behind complex phenotype such as aging and ASLI.

## Author Contributions

RU and SMS conceived and designed the experiments; contributed materials and analysis tools, and wrote this article. RU performed the experiments and analyzed the data.

## Conflict of Interest Statement

The authors declare that the research was conducted in the absence of any commercial or financial relationships that could be construed as a potential conflict of interest.

## Footnotes

^1^http://www.ingenuity.com

^2^http://david.abcc.ncifcrf.gov/
