# Supplementary material for: Gene Network Construction from Microarray Data Identifies a Key Network Module and Several Candidate Hub Genes in Age-Associated Spatial Learning Impairment
Source: Front Syst Neurosci. 2017 Oct 10;11:75. doi: 10.3389/fnsys.2017.00075 (PMC5641338; doi:10.3389/fnsys.2017.00075)
Supplement: Supplementary file 1 [file Data_Sheet_1.docx]

*Supplementary Material*

# Gene network construction from microarray data identifies a key network module and several candidate hub genes in age-associated spatial learning impairment

Raihan Uddin* and Shiva M. Singh

***Correspondence:** Raihan Uddin, [ruddin@uwo.ca](mailto:ruddin@uwo.ca)

**1 Supplementary Figures and Tables**

**1.1 Supplementary Figures**

**
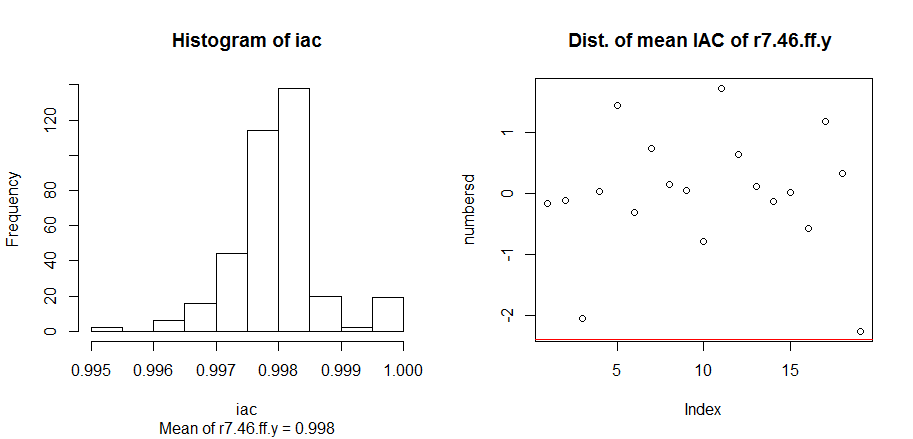

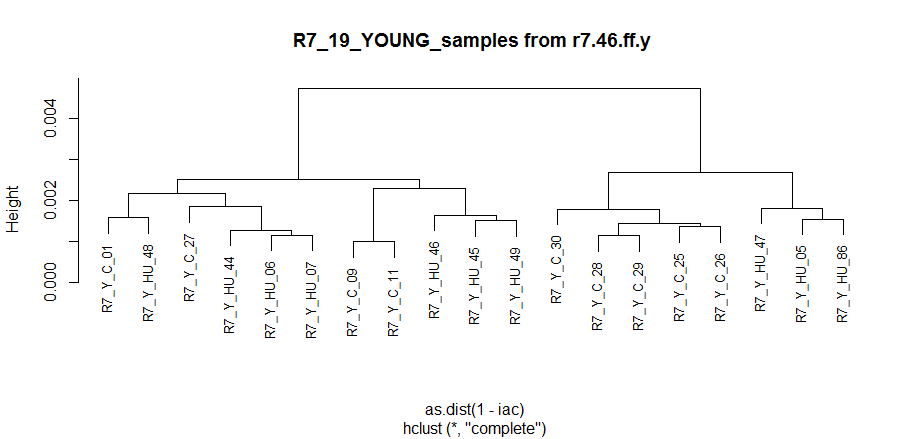
**

**A**

**B**

**C**

**Supplementary Figure S1. IAC based quality check for R7 young dataset.** The mean IAC for the 19 young samples were 0.998 (A) and all arrays were 2.5 standard deviations below the mean (B). No outlier is evident in the hierarchical clustering dendrogram (C).


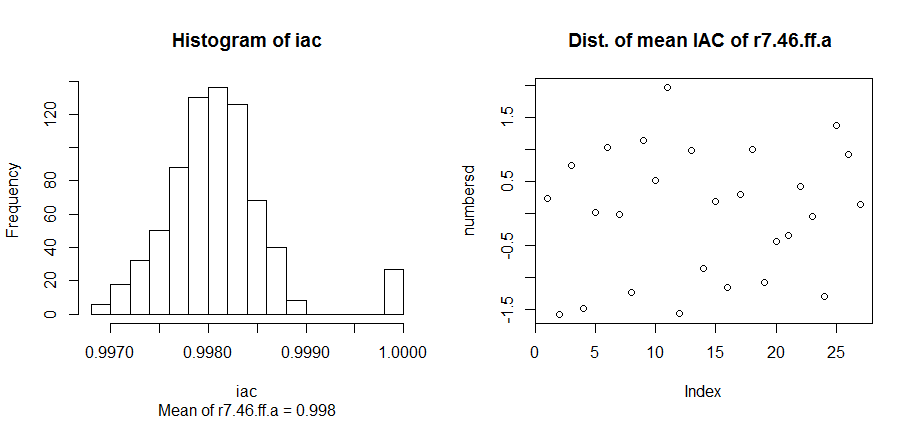

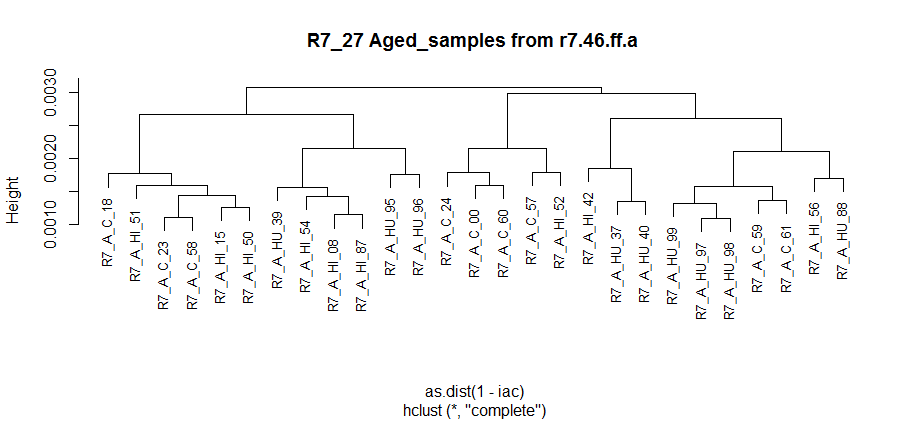


**A**

**B**

**C**

**Supplementary Figure S2. IAC based quality check for R7 aged dataset.** The mean IAC for the 27 aged samples were 0.998 (A) and all arrays were 2 standard deviations below the mean (B). No outlier is evident in the hierarchical clustering dendrogram (C).


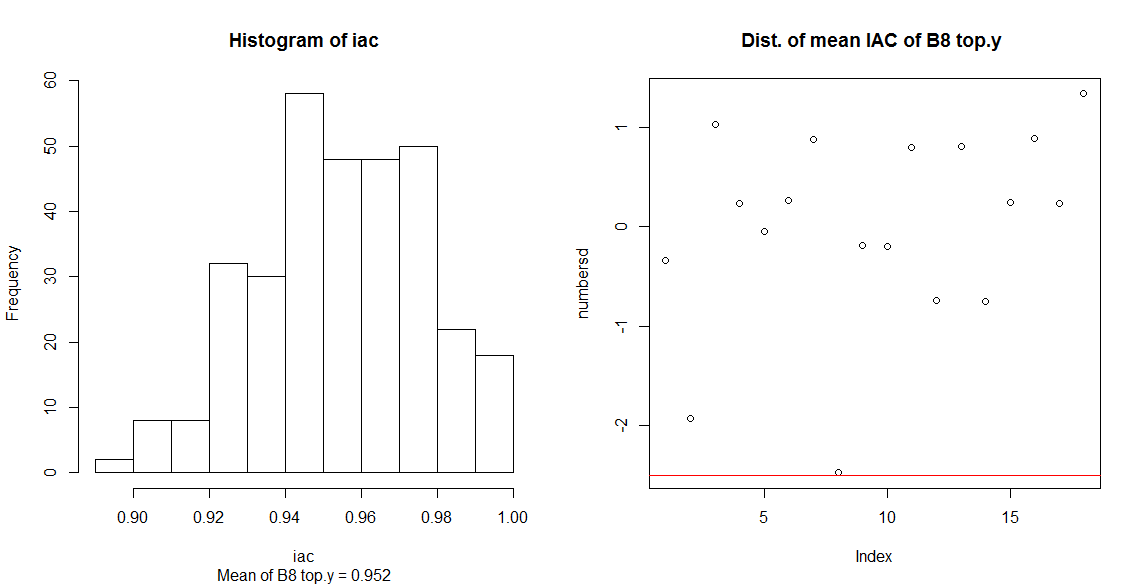


**A**

**B**

**C**


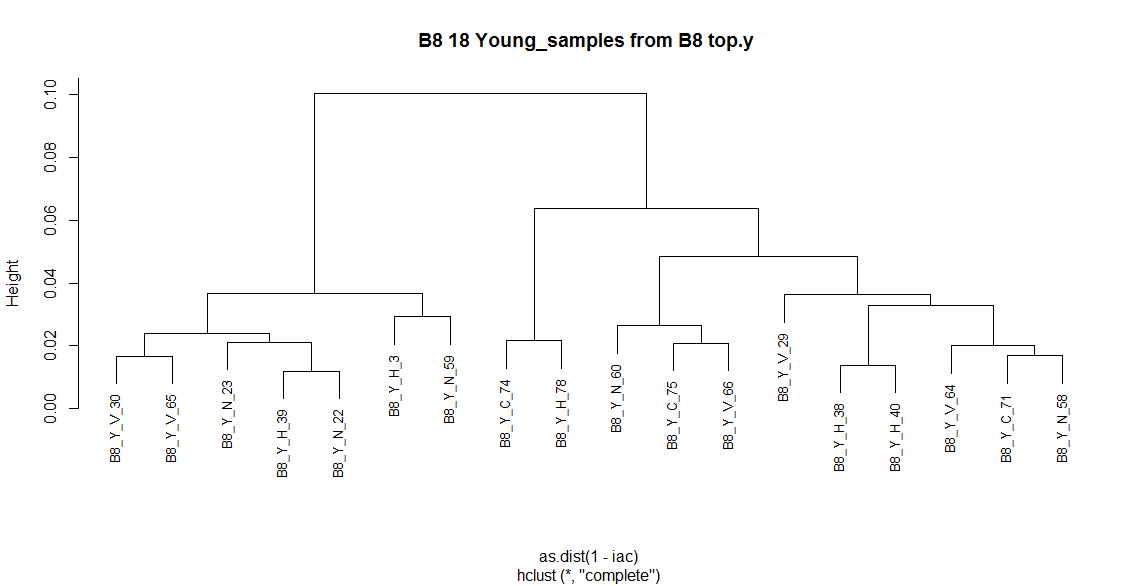


**Supplementary Figure S3. IAC based quality check for B8 young dataset.** The mean IAC for the 18 young samples were 0.952 (A) and all arrays were within 3 standard deviations below the mean (B). No outlier is evident in the hierarchical clustering dendrogram (C).


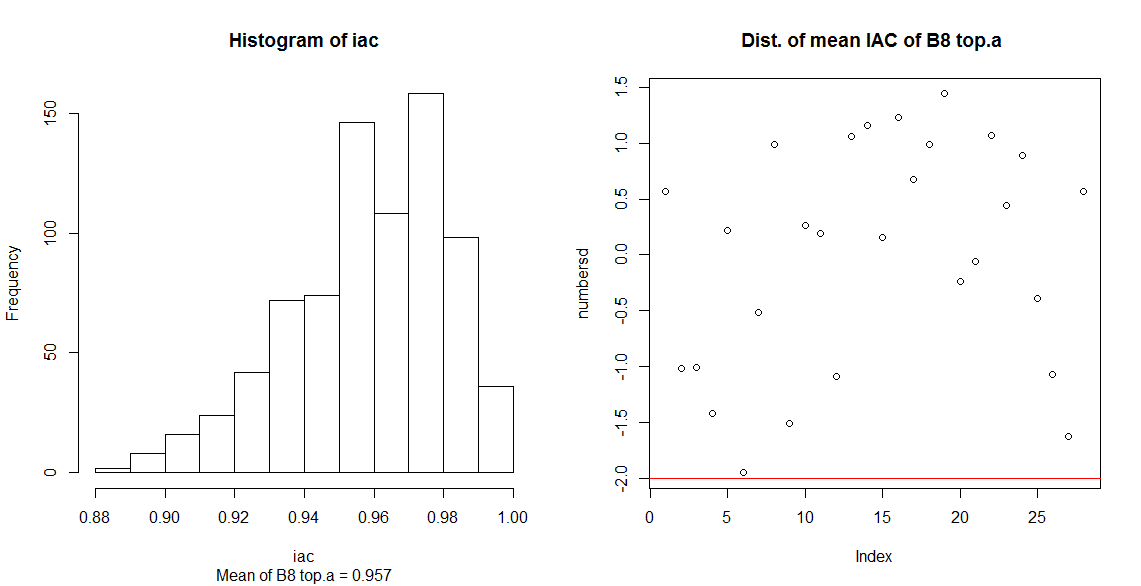


**A**

**B**

**C**


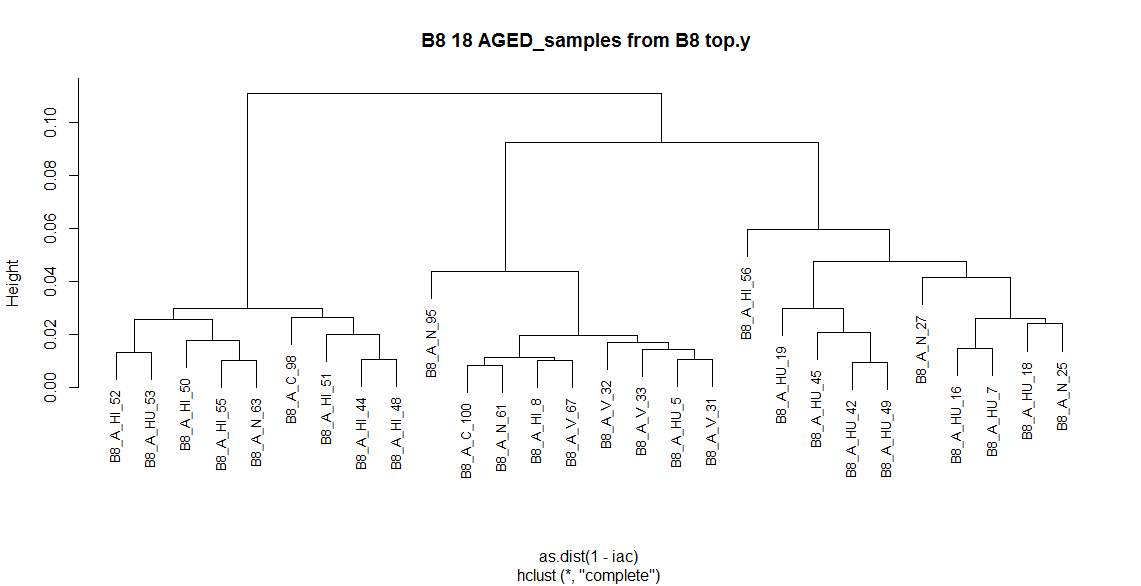


**Supplementary Figure S4. IAC based quality check for B8 aged dataset.** The mean IAC for the 28 aged samples were 0.957 (A) and all arrays were 2 standard deviations below the mean (B). No outlier is evident in the hierarchical clustering dendrogram (C).


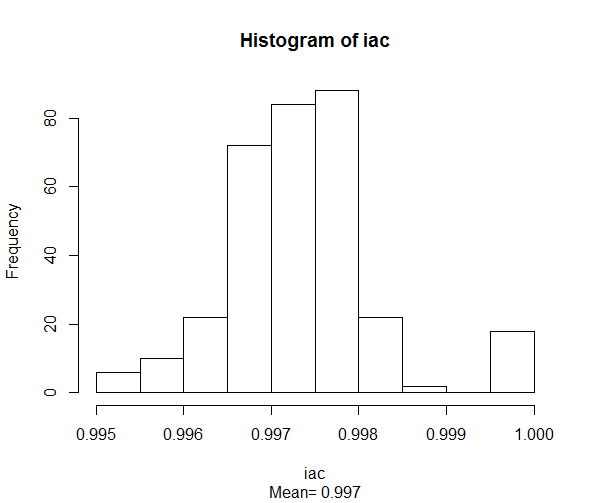

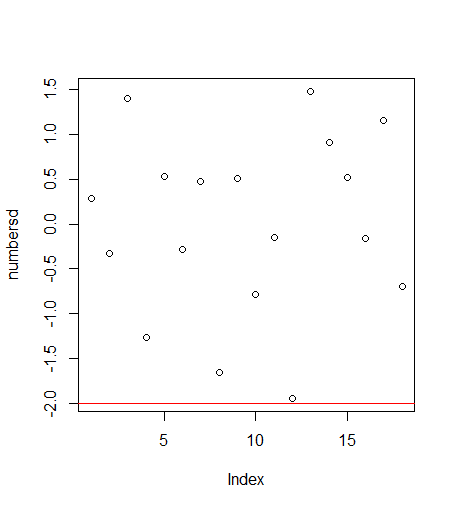

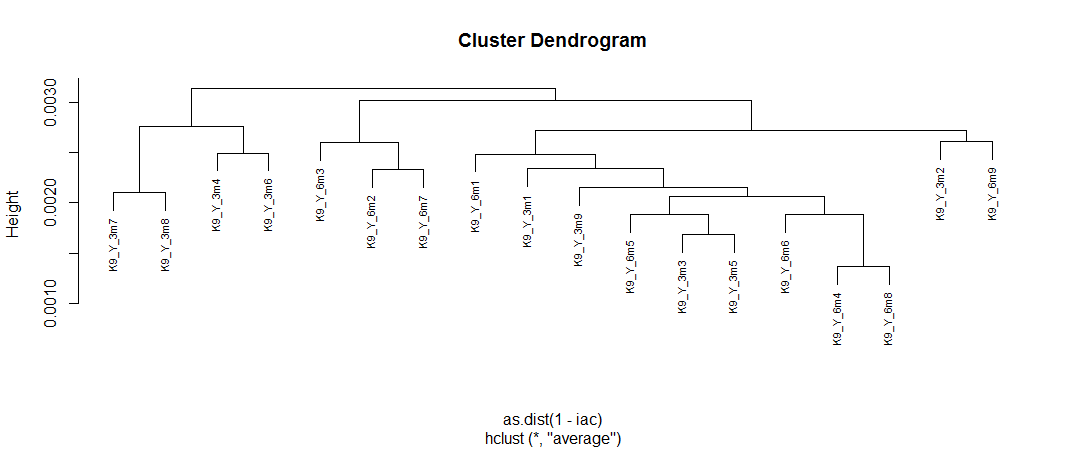


**A**

**B**

**C**

**Dist. of mean IAC of K9-Y**

**Supplementary Figure S5. IAC based quality check for K9 young dataset.** The mean IAC for the 18 young samples were 0.997 (A) and all arrays were 2 standard deviations below the mean (B). No outlier is evident in the hierarchical clustering dendrogram (C).


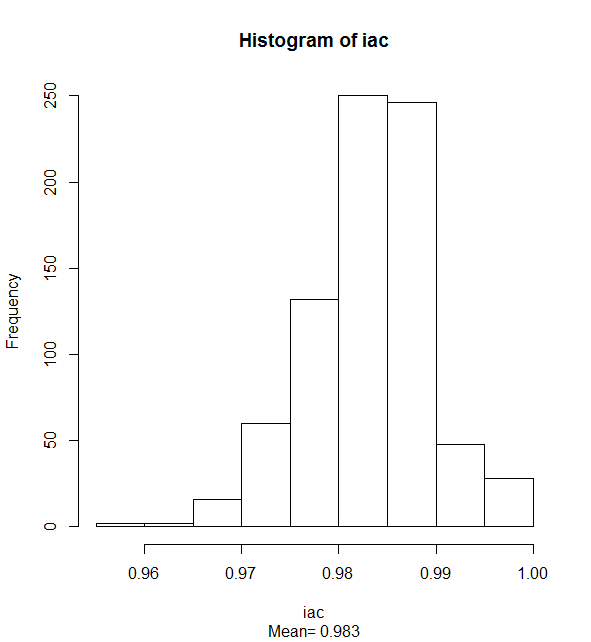

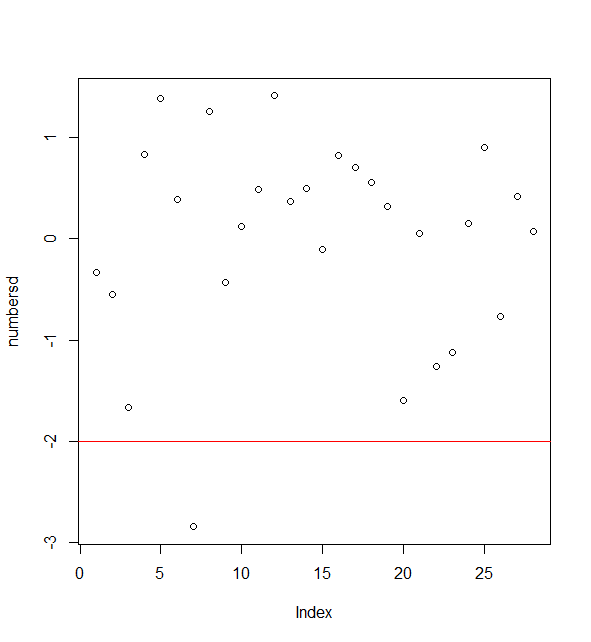


**Dist. of mean IAC of B7-A**

**A**

**B**

**C**


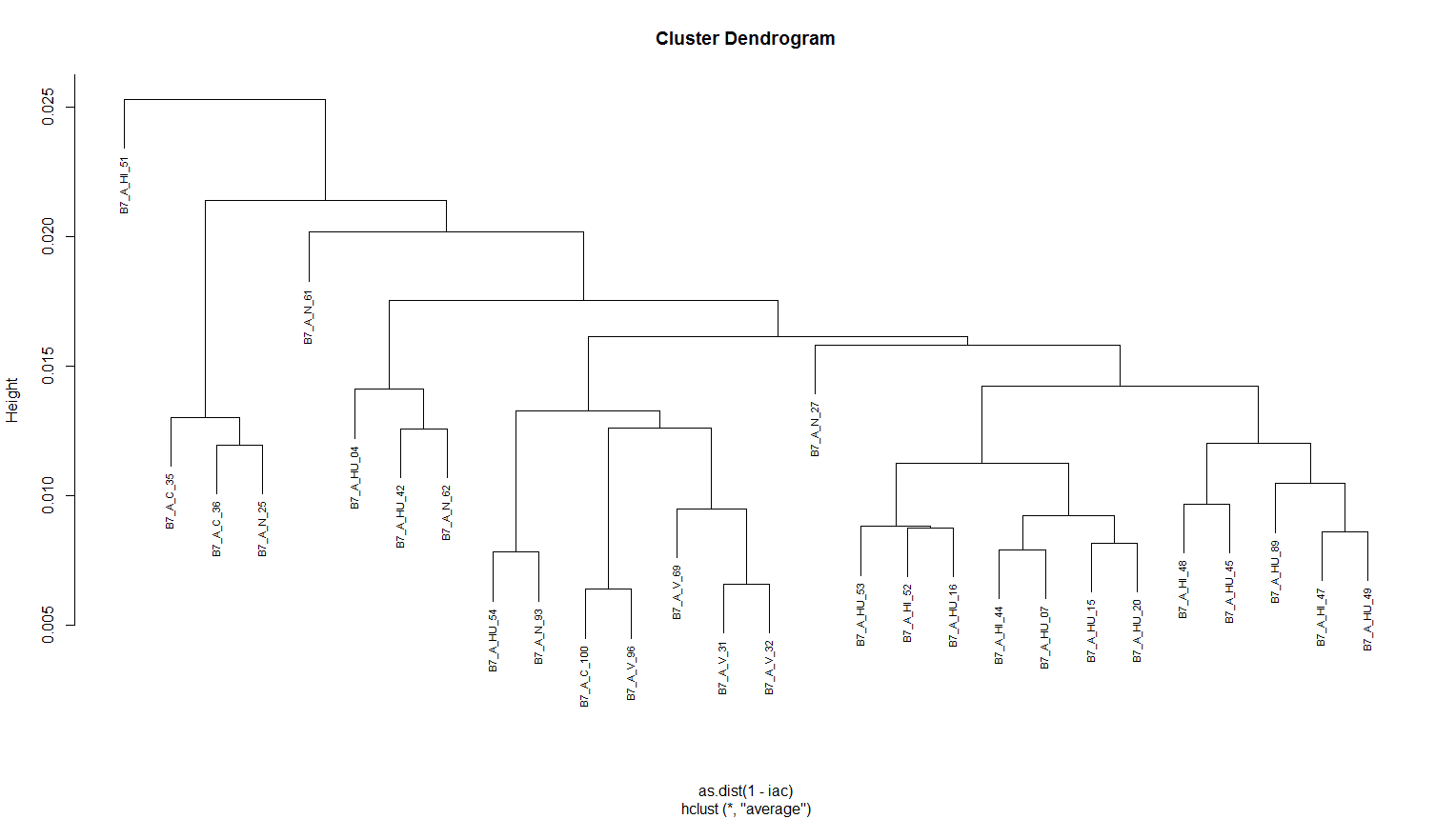


**Supplementary Figure S6. IAC based quality check for B7 aged dataset.** The mean IAC for the 28 young samples were 0.983 (A) and all arrays were 3 standard deviations below the mean (B). No outlier is evident in the hierarchical clustering dendrogram (C).


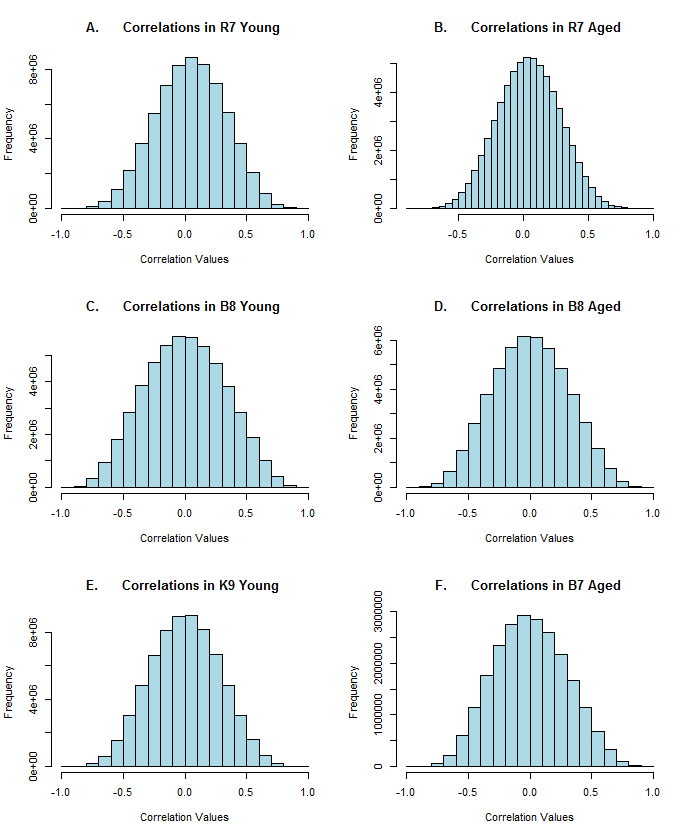


**Supplementary Figure S7. Histogram of correlations between genes in each dataset selected for WGCNA network analysis.**  Correlations are centered at zero for R7-Y (A), B8-Y (C), B8-A (D), K9-Y (E), and B7-A (F), and close to zero for R7-A (B). Thus the correlations are reasonable as they are mostly centered at zero.


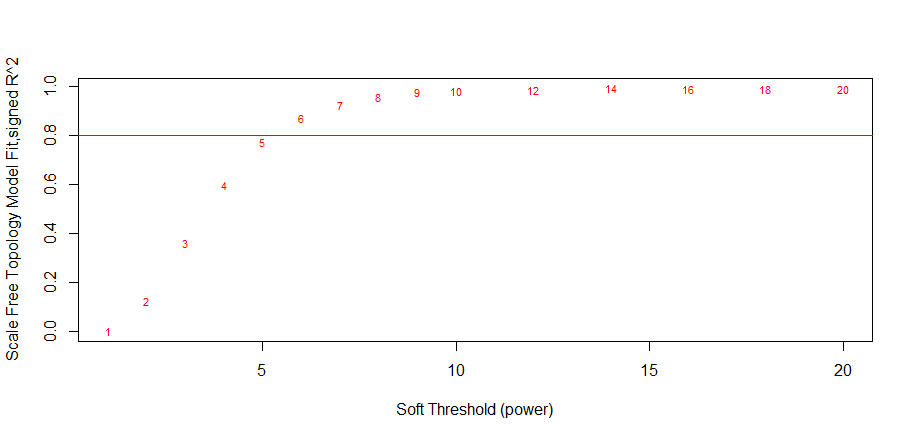


A **Scale-free fit index as a function of beta**

B **Scale free topology plot**


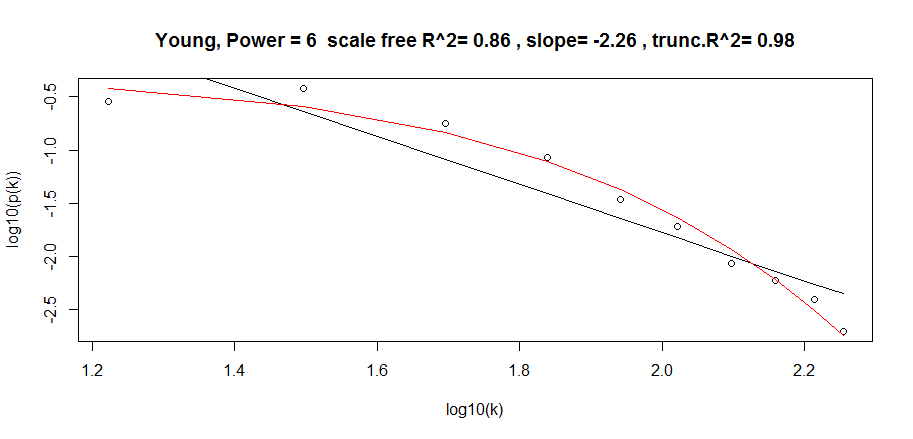


**Supplementary Figure S8. Analysis of network topology for various soft-thresholding powers for the R7 young dataset.** A) Scale-free fit index (y-axis) as a function of the soft-thresholding power (x-axis). B) Scale free topology plot shows the log-log plot between frequency of connectivity p(k) and connectivity k for determining whether the network exhibits scale-free topology.


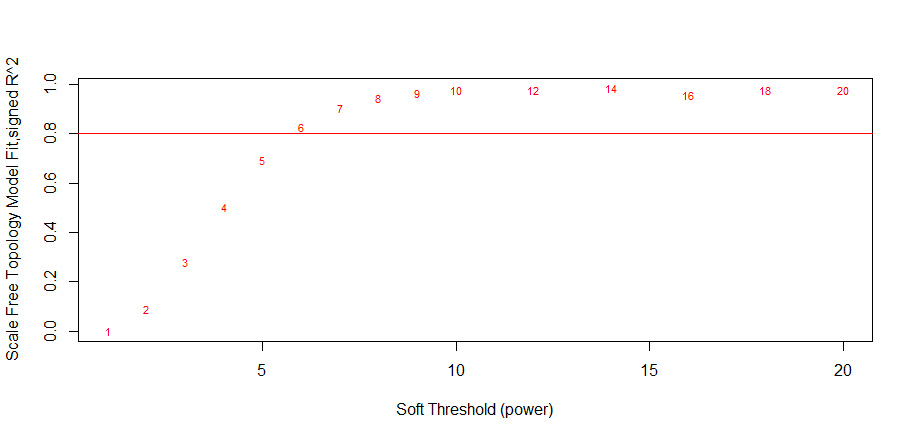


A **Scale-free fit index as a function of beta**

B **Scale free topology plot**


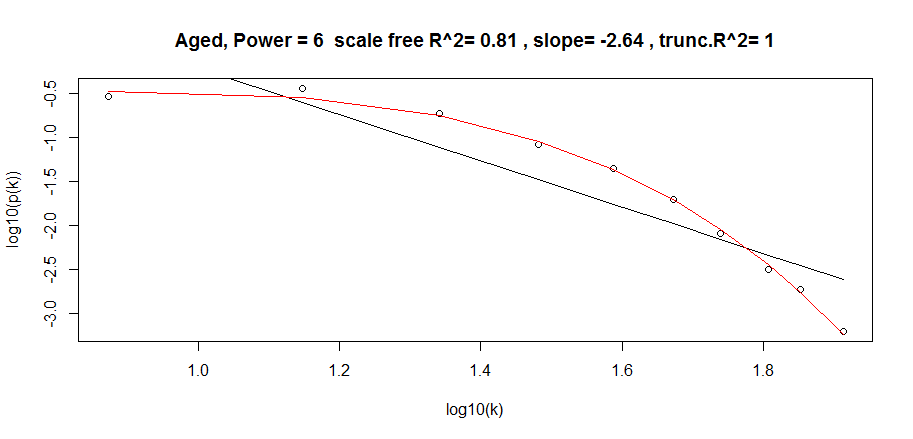


**Supplementary Figure S9. Analysis of network topology for various soft-thresholding powers for the R7 aged dataset.** A) Scale-free fit index (y-axis) as a function of the soft-thresholding power (x-axis). B) Scale free topology plot shows the log-log plot between frequency of connectivity p(k) and connectivity k for determining whether the network exhibits scale-free topology.


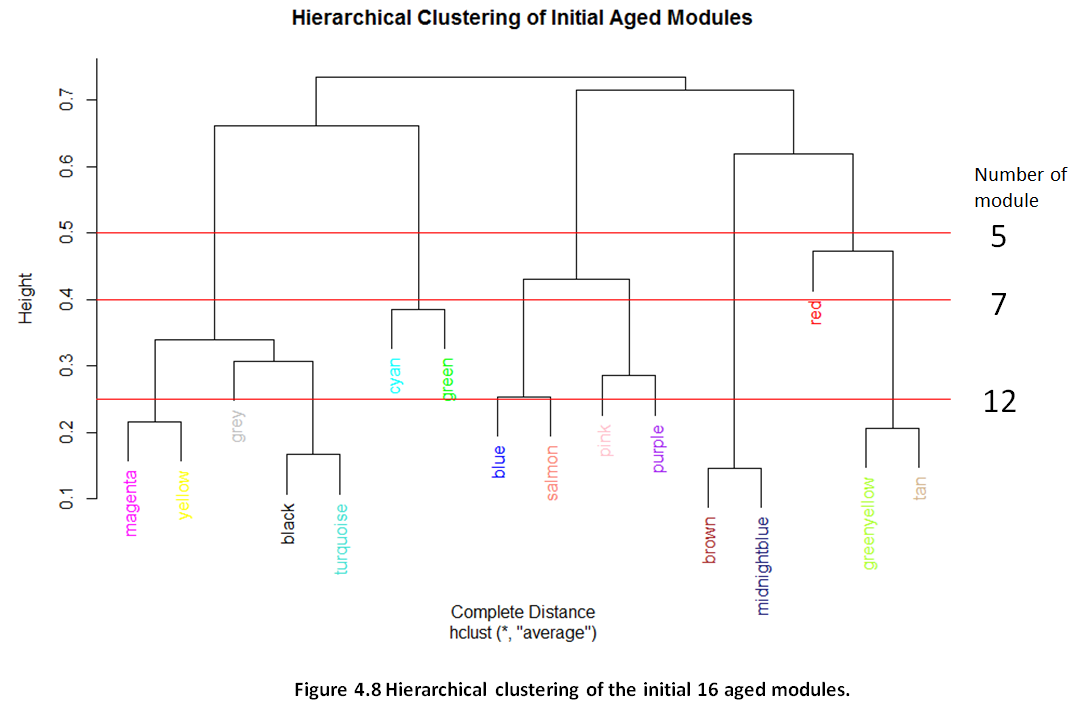


**Supplementary Figure S10.** **An average linkage hierarchical plot of the module eigengenes of the 16 aged modules (including the grey module)**. It shows that some modules (e.g. the black and turquoise, cyan and green, blue and salmon, etc.) are clustered very close together. The three red lines represent the tree cut line at different heights and the numbers on the right (corresponding to the lines) represent the expected number of resulting merged modules that each cut will produce. The modules that are clustered close together are merged into new modules (Figure 3), for example, the magenta, yellow, black, and turquoise modules are merged into a new module named black; cyan and green are merged into cyan; blue and salmon are merged into blue; pink and purple are merged into pink; brown and midnightblue are merged into brown; and red, greenyellow, and tan modules are merged into red. The grey module contained genes that did not belong to any module and remained separate.

**
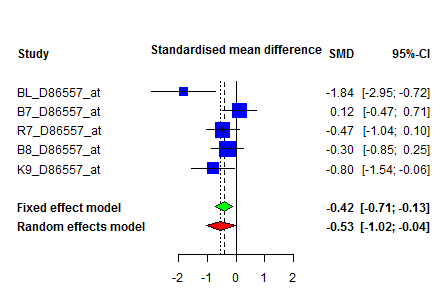
**

**Supplementary Figure S11. Forest plot of *Camk1g*.** For the selected probe set for this gene the individual study specific SMDs and their 95% confidence intervals are plotted and shown on each row. The effect size results are shown at the bottom of the plot.

**
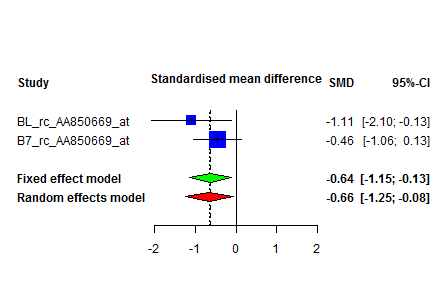
**

**Supplementary Figure S12. Forest plot of *Cdk5r1*.**  For the selected probe set for this gene the individual study specific SMDs and their 95% confidence intervals are plotted and shown on each row. The effect size results are shown at the bottom of the plot.

**
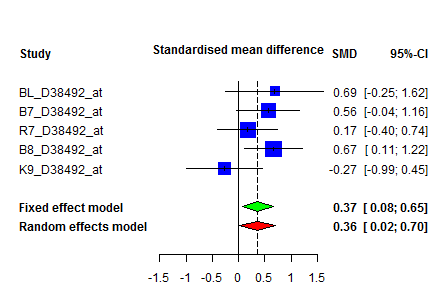
**

**Supplementary Figure S13. Forest plot of *Cntn1*.**  For the selected probe set for this gene the individual study specific SMDs and their 95% confidence intervals are plotted and shown on each row. The effect size results are shown at the bottom of the plot. *Cntn1* was up regulated in the aged rats (effect size = +0.36, pvalue = 0.04) in my combined meta-analysis with the K9 study showing a down regulation.

**
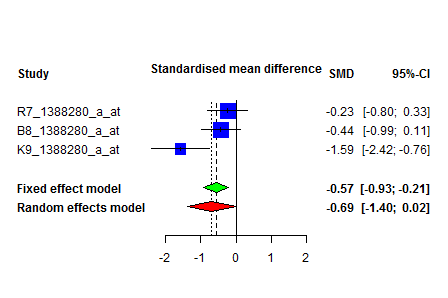
**

**Supplementary Figure S14. Forest plot of *Dlg3*.**  For the selected probe set for this gene the individual study specific SMDs and their 95% confidence intervals are plotted and shown on each row. The effect size results are shown at the bottom of the plot. *Dlg3* showed lower expression (effect size = -0.69, p-value 0.06) in the aged rats compared to the young in three of the five studies

**
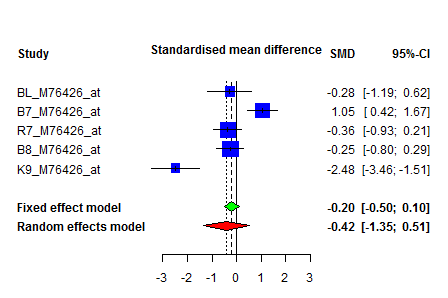
**

**Supplementary Figure S15. Forest plot of *Dpp6*.**  For the selected probe set for this gene the individual study specific SMDs and their 95% confidence intervals are plotted and shown on each row. The effect size results are shown at the bottom of the plot. *Dpp6* is another hub gene that showed lower expression in the aged compared to young in the meta-analysis in 4 of the 5 studies (effect size = -0.42, p-value = 0.38), with only B7 showing higher expression in the aged.

**
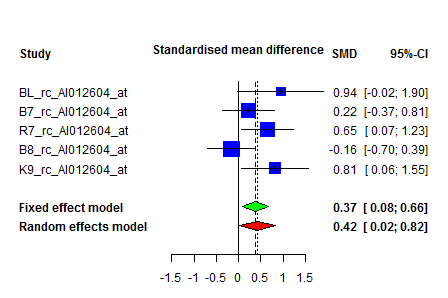
**

**Supplementary Figure S16. Forest plot of *Eif5*.**  For the selected probe set for this gene the individual study specific SMDs and their 95% confidence intervals are plotted and shown on each row. The effect size results are shown at the bottom of the plot.

**
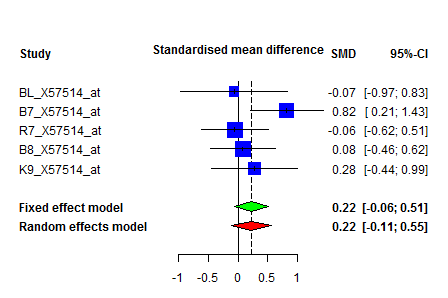
**

**Supplementary Figure S17. Forest plot of *Gabrg1*.** For the selected probe set for this gene the individual study specific SMDs and their 95% confidence intervals are plotted and shown on each row. The effect size results are shown at the bottom of the plot.

**
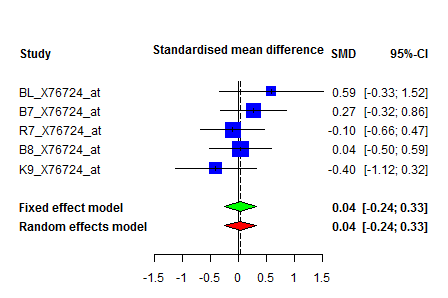
**

**Supplementary Figure S18. Forest plot of *Kcnab2*.** For the selected probe set for this gene the individual study specific SMDs and their 95% confidence intervals are plotted and shown on each row. The effect size results are shown at the bottom of the plot. *Kcnab2* showed an effect size of 0.04 with a p-value of 0.77. This is due to the fact that the SMD was slightly down in the aged in R7 and K9, but up in BL, B7 and B8. Given the diverse and delicate nature of these ion channels, which are constantly changing in quantity and locations, none of the studies was successful in recording the exact expression of this gene.

**
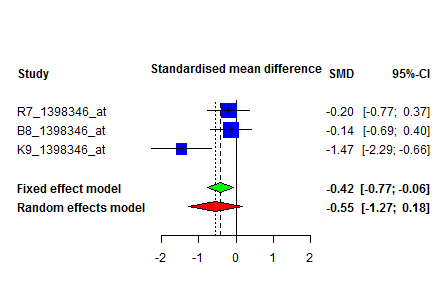
**

**Supplementary Figure S19. Forest plot of *Mapk1*.** For the selected probe set for this gene the individual study specific SMDs and their 95% confidence intervals are plotted and shown on each row. The effect size results are shown at the bottom of the plot. *Mapk1*, a learning gene, and was down regulated in the aged (effect size = -0.55. p-value = 0.14).

**
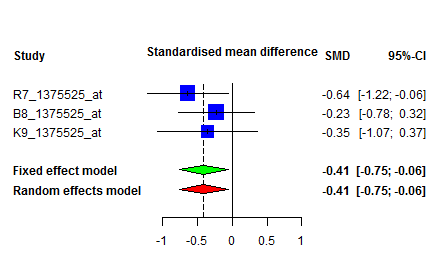
**

**Supplementary Figure S20. Forest plot of *Mapre1*.**  For the selected probe set for this gene the individual study specific SMDs and their 95% confidence intervals are plotted and shown on each row. The effect size results are shown at the bottom of the plot. Down-regulation of *Mapre1* (*EB1*) in the aged rats (effect size = -0.41, p-value = 0.02) and the associated deficiency in learning of these aged rats is in line with the findings in the literature.

**
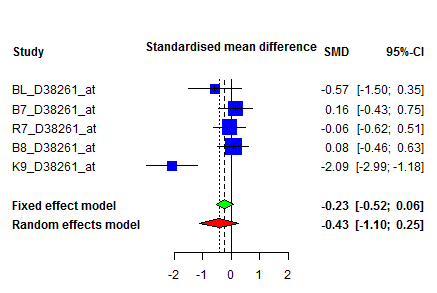
**

**Supplementary Figure S21. Forest plot of *Ppp2r2c*.**  For the selected probe set for this gene the individual study specific SMDs and their 95% confidence intervals are plotted and shown on each row. The effect size results are shown at the bottom of the plot. *Ppp2r2c* was down-regulated in the aged rats (effect size -0.43, p-value 0.22).

**
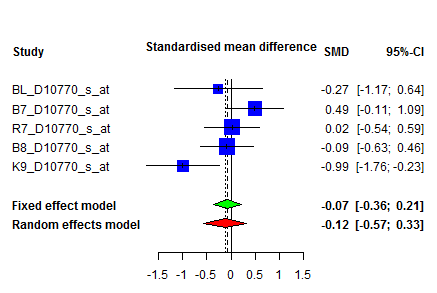
**

**Supplementary Figure S22. Forest plot of *Prkacb*.**  For the selected probe set for this gene the individual study specific SMDs and their 95% confidence intervals are plotted and shown on each row. The effect size results are shown at the bottom of the plot. The *Cbeta* gene was down-regulated in the aged rats compared to the young with an effect size of -0.1214 and p-value of 0.59.

**
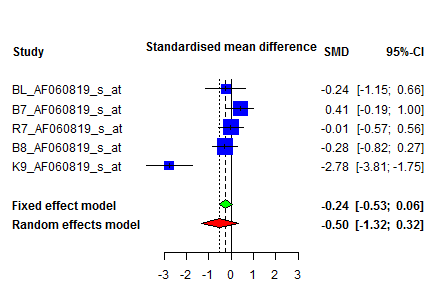
**

**Supplementary Figure S23. Forest plot of *Rasgrp1*.**  For the selected probe set for this gene the individual study specific SMDs and their 95% confidence intervals are plotted and shown on each row. The effect size results are shown at the bottom of the plot. *Rasgrp1* was down-regulated in the aged with an effect size of -0.50 and p-value of 0.23 in 4 of the 5 studies assessed in this meta-analysis.

**
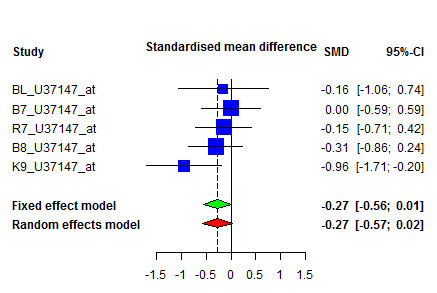
**

**Supplementary Figure S24. Forest plot of *Scn2b*.**  For the selected probe set for this gene the individual study specific SMDs and their 95% confidence intervals are plotted and shown on each row. The effect size results are shown at the bottom of the plot.

**
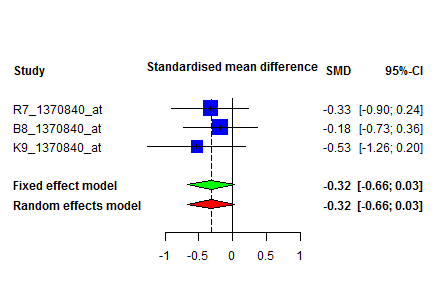
**

**Supplementary Figure S25. Forest plot of *Stxbp1*.**  For the selected probe set for this gene the individual study specific SMDs and their 95% confidence intervals are plotted and shown on each row. The effect size results are shown at the bottom of the plot. *Stxbp1*, also known as *Munc18-1*, is down-regulated in the aged rats (effect size = -0.32, p-value = 0.07)


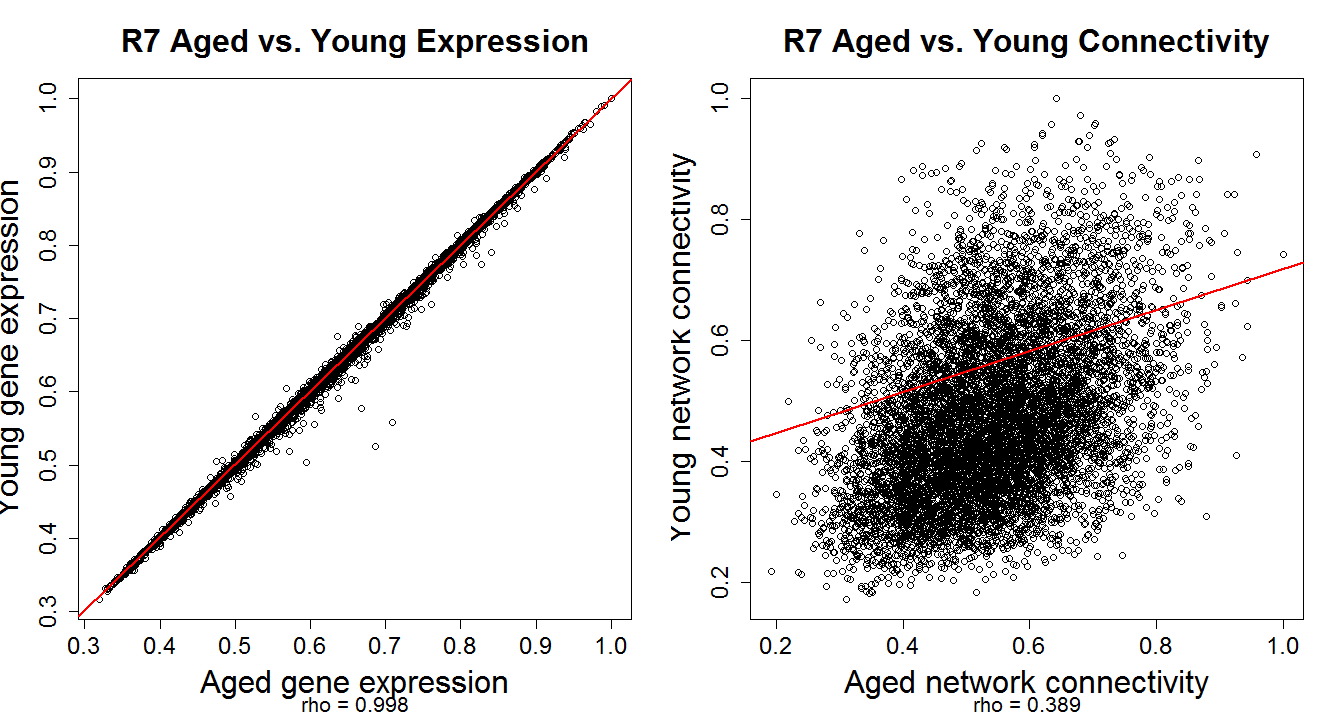


**Supplementary Figure S26. An example of differential expression vs. differential connectivity.** This figure compares gene expression and connectivity between young and aged R7 samples using scatter plots. Mean gene expressions for each gene across all aged and young arrays were calculated. The gene expression values were then scaled to lie between 0 and 1 by dividing them with the maximum mean expression. The young and aged mean scaled gene expression values were plotted in the scatter plot (on the left image below) along the y and x axis, respectively. Similarly, connectivity for each gene was calculated, scaled to lie between 0 and 1, and plotted on the right scatter plot. Spearman’s rank correlation test (rho) was performed between young vs. aged gene expression as well as young vs. aged network connectivity. The results show that the overall gene expressions between the young and aged samples are highly correlated (rho = 0.998), which is not the case for the gene networks connectivity patterns between the same samples as the correlations between them are very weak (rho = 0.389). This observation highlights the fact that differential connectivity is not the same as differential expression.

**1.2 Supplementary Tables**

**Supplementary Table S1. Number of arrays selected from each datasets after preprocessing.**

| Study name | Original number of arrays | Final number of arrays after quality control and outlier removal | | |
| --- | --- | --- | --- | --- |
|  |  | Total | Young (Y) | Aged (A) |
| B7 | 79 | 38 | 10 | 28 |
| R7 | 50 | 46 | 19 | 27 |
| B8 | 80 | 46 | 18 | 28 |
| K9 | 49 | 30 | 18 | 12 |
| BL | 29 | 15 | 8 | 7 |

**Supplementary Table S2. Gene selection for network comparison.** This table shows the number of genes that remained for network analysis and comparison after low-connectivity gene filtering, and after matching the R7 young module labels to the B8, K9, and B7 data. For B7, no low-connectivity filtering was done because of the already low number of remaining genes.

|  | Number of genes remained for network analysis | | | | |
| --- | --- | --- | --- | --- | --- |
| Dataset | After preprocessing | Median Connectivity (kmed) | After low-connectivity gene filtering | Module overlap comparison | After matching R7 young genes |
| R7 | 8053 | 0.5 | 5674 |  | -- |
| B8 | 7157 | 0.4 | 5202 | R7-Y vs. B8-Y  R7-A vs. B8-A | 3626 |
| K9 | 8250 | 0.35 | 4796 | R7-Y vs. K9-Y | 3138 |
| B7 | 4829 | - - - | 4829 | R7-A vs. B7-A | 2140 |

**Supplementary Table S3. R7-Y to R7-A overlap table. Numbers in bold represent the genes that match between each pair of modules with the most significant p-value given in Supplementary Table S4.**

| Aged  Young | Black (pink) | Brown (black) | Green (brown) | grey | Red (red) | Turquoise (blue) | Yellow (cyan) |
| --- | --- | --- | --- | --- | --- | --- | --- |
| blue | 54 | **350** | 39 | 499 | 28 | 40 | 5 |
| brown | 21 | **308** | 84 | 314 | 9 | 11 | 12 |
| green | 6 | 72 | **104** | 162 | 12 | 18 | 6 |
| grey | 113 | 159 | 113 | 660 | 51 | 218 | 5 |
| red | 11 | 21 | 91 | 142 | **63** | 13 | 0 |
| turquoise | **135** | 188 | 54 | 541 | 37 | **173** | 1 |
| yellow | 26 | 53 | 69 | 282 | 6 | 35 | **260** |

**Supplementary Table S4. R7-Y to R7-A overlap p-value table. Values in bold represent the most significant p-values of the corresponding matches between each pair of modules shown in Supplementary Table S3.**

| Aged    Young | Black (pink) | Brown (black) | Green (brown) | grey | Red (red) | Turquoise (blue) | Yellow (cyan) |
| --- | --- | --- | --- | --- | --- | --- | --- |
| blue | 9.57E-01 | **2.98E-32** | 1.00E+00 | 1.02E-02 | 9.62E-01 | 1.00E+00 | 1.00E+00 |
| brown | 1.00E+00 | **5.41E-44** | 1.10E-01 | 9.96E-01 | 1.00E+00 | 1.00E+00 | 1.00E+00 |
| green | 1.00E+00 | 7.68E-01 | **1.31E-24** | 9.11E-01 | 7.36E-01 | 1.00E+00 | 1.00E+00 |
| grey | 3.22E-04 | 1.00E+00 | 9.59E-01 | 2.59E-04 | 3.26E-01 | 4.20E-25 | 1.00E+00 |
| red | 9.98E-01 | 1.00E+00 | 1.18E-20 | 9.51E-01 | **1.93E-29** | 1.00E+00 | 1.00E+00 |
| turquoise | **3.90E-15** | 1.00E+00 | 1.00E+00 | 6.12E-02 | 7.86E-01 | **2.42E-15** | 1.00E+00 |
| yellow | 1.00E+00 | 1.00E+00 | 6.45E-01 | 1.00E+00 | 1.00E+00 | 1.00E+00 | **9.10E-214** |

**Supplementary Table S5. Meta-Analysis effect size estimates of top candidate ASLI hub genes in R7 (yellow) “learning and memory” module.**

| Gene | Probe ID (RGU34A or RAE230A) | Rat Gene ID | Number | ES | z-value | p-value of z-value | pBH of z-value | Q value | p-value of Q | tau.2 | I2 |
| --- | --- | --- | --- | --- | --- | --- | --- | --- | --- | --- | --- |
| Symbol |  |  | Of Study |  |  |  |  |  |  |  |  |
| Camk1g | D86557_at | 171358 | 5 | -0.53 | -2.10 | 0.04 | 0.19 | 10.63 | 0.03 | 0.19 | 62.40 |
| Cdk5r1 | rc_AA850669_at | 116671 | 2 | -0.66 | -2.21 | 0.03 | 0.17 | 1.22 | 0.27 | 0.04 | 18.10 |
| Cntn1 | D38492_at | 117258 | 5 | 0.36 | 2.09 | 0.04 | 0.20 | 5.46 | 0.24 | 0.04 | 26.70 |
| Dlg3 | 1388280_a_at | 58948 | 3 | -0.69 | -1.92 | 0.06 | 0.25 | 7.35 | 0.03 | 0.28 | 72.80 |
| Dlgap1 | U67987_s_at | 65040 | 5 | -0.12 | -0.47 | 0.64 | 0.80 | 12.35 | 0.01 | 0.23 | 67.60 |
| Dpp6 | M76426_at | 29272 | 5 | -0.42 | -0.88 | 0.38 | 0.64 | 36.78 | 0.00 | 0.99 | 89.10 |
| Eif5 | rc_AI012604_at | 56783 | 5 | 0.42 | 2.05 | 0.04 | 0.21 | 7.41 | 0.12 | 0.09 | 46.00 |
| Gabrg1 | X57514_at | 140674 | 5 | 0.22 | 1.32 | 0.19 | 0.46 | 5.25 | 0.26 | 0.03 | 23.90 |
| Impact | 1375310_at | 497198 | 3 | 0.41 | 2.28 | 0.02 | 0.15 | 0.53 | 0.77 | 0.00 | 0.00 |
| Kcnab2 | X76724_at | 29738 | 5 | 0.04 | 0.29 | 0.77 | 0.88 | 3.61 | 0.46 | 0.00 | 0.00 |
| Mapk1 | 1398346_at | 116590 | 3 | -0.55 | -1.47 | 0.14 | 0.41 | 7.98 | 0.02 | 0.31 | 74.90 |
| Mapre1 | 1375525_at | 114764 | 3 | -0.41 | -2.28 | 0.02 | 0.15 | 1.04 | 0.60 | 0.00 | 0.00 |
| Ndfip2 | 1389364_at | 361089 | 3 | -0.38 | -1.22 | 0.22 | 0.50 | 5.84 | 0.05 | 0.19 | 65.70 |
| Ppp2r2c | D38261_at | 117256 | 5 | -0.43 | -1.24 | 0.22 | 0.50 | 20.01 | 0.00 | 0.46 | 80.00 |
| Prkacb | D10770_s_at | 293508 | 5 | -0.12 | -0.53 | 0.60 | 0.78 | 9.36 | 0.05 | 0.15 | 57.30 |
| Pten | rc_AA963447_at | 50557 | 5 | -0.37 | -2.58 | 0.01 | 0.09 | 1.49 | 0.83 | 0.00 | 0.00 |
| Rasgrp1 | AF060819_s_at | 29434 | 5 | -0.50 | -1.20 | 0.23 | 0.51 | 28.63 | 0.00 | 0.73 | 86.00 |
| Scn2b | U37147_at | 25349 | 5 | -0.28 | -1.84 | 0.07 | 0.28 | 4.21 | 0.38 | 0.01 | 4.90 |
| Stxbp1 | 1370840_at | 25558 | 3 | -0.32 | -1.79 | 0.07 | 0.29 | 0.57 | 0.75 | 0.00 | 0.00 |

Legends: ES, effect size; pBH, p-value with Benjamini and Hochberg correction; FC, fold change; DE, differentially expressed; Q = Cochran's Q test for significant heterogeneity; I^2 = Ratio of true heterogeneity to total variation.

Supplementary Table S6. Repeatability of young R7 yellow module hub genes in B8 young matching (red and brown) modules. Twenty hub genes with the highest mean $\boldsymbol{k}_{\boldsymbol{IM}}$ are shown. Candidate hub genes are marked by an ‘*’ beside them.

| Hub Gene | R7 KME | B8 KME | Mean KME | t-test p-value |
| --- | --- | --- | --- | --- |
| Dlgap1* | 0.98 | 0.69 | 0.83 | 0.11 |
| Fkbp1a | 0.88 | 0.73 | 0.80 | 0.06 |
| Rab3a | 0.75 | 0.82 | 0.79 | 0.03 |
| Ppp4r2 | 0.74 | 0.83 | 0.79 | 0.04 |
| Xpr1 | 0.84 | 0.71 | 0.78 | 0.05 |
| Glul | 0.86 | 0.68 | 0.77 | 0.08 |
| Dlg3* | 0.99 | 0.51 | 0.75 | 0.20 |
| Stxbp1* | 0.96 | 0.52 | 0.74 | 0.18 |
| Sri | 0.86 | 0.61 | 0.74 | 0.10 |
| Got1 | 0.78 | 0.68 | 0.73 | 0.04 |
| Zfp292 | 0.49 | 0.96 | 0.73 | 0.20 |
| Psme4 | 0.84 | 0.55 | 0.70 | 0.13 |
| Cacng3 | 0.71 | 0.65 | 0.68 | 0.03 |
| Mapre1* | 0.99 | 0.35 | 0.67 | 0.28 |
| Cnpy2 | 0.56 | 0.78 | 0.67 | 0.11 |
| Nsf | 0.95 | 0.36 | 0.65 | 0.27 |
| Dpp6* | 0.93 | 0.38 | 0.65 | 0.26 |
| Arfgap1 | 0.62 | 0.67 | 0.65 | 0.03 |
| Odc1 | 0.38 | 0.91 | 0.65 | 0.25 |
| Pafah1b2 | 0.73 | 0.55 | 0.64 | 0.09 |

Supplementary Table S7. Repeatability of young R7 yellow module hub genes in young K9 matching (brown and yellow) modules. Twenty hub genes with the highest mean $\boldsymbol{k}_{\boldsymbol{IM}}$ are shown. Candidate hub genes are marked by an ‘*’ beside them.

| Hub Gene | R7 KME | K9 KME | Mean KME | t-test p-value |
| --- | --- | --- | --- | --- |
| Scn2b* | 0.93 | 0.93 | 0.93 | 0.00 |
| Prkacb* | 1.00 | 0.85 | 0.92 | 0.05 |
| Pclo | 0.88 | 0.92 | 0.90 | 0.01 |
| Dctn4 | 0.85 | 0.87 | 0.86 | 0.01 |
| Cacnb4* | 0.78 | 0.93 | 0.86 | 0.06 |
| Ndfip2* | 0.87 | 0.81 | 0.84 | 0.02 |
| Mtpn | 0.96 | 0.72 | 0.84 | 0.09 |
| Cntn1* | 0.86 | 0.81 | 0.83 | 0.02 |
| Impact* | 0.97 | 0.68 | 0.83 | 0.11 |
| Dnal1 | 0.92 | 0.71 | 0.82 | 0.08 |
| Pten* | 0.78 | 0.86 | 0.82 | 0.03 |
| G3bp2 | 0.88 | 0.75 | 0.81 | 0.05 |
| Dnm1l | 0.94 | 0.68 | 0.81 | 0.10 |
| Trim23 | 0.89 | 0.69 | 0.79 | 0.08 |
| Ranbp2 | 0.84 | 0.72 | 0.78 | 0.05 |
| Akap6 | 0.95 | 0.60 | 0.77 | 0.14 |
| Tmem30a | 0.76 | 0.77 | 0.77 | 0.00 |
| Fam91a1 | 0.59 | 0.94 | 0.77 | 0.14 |
| Atf2 | 0.78 | 0.75 | 0.77 | 0.01 |
| Arl1 | 0.81 | 0.70 | 0.76 | 0.05 |

Supplementary Table S8. Repeatability of young R7 yellow module hub genes in young B8 matching (brown and red), and young K9 matching (brown and yellow) modules. Twenty hub genes with the highest mean $\boldsymbol{k}_{\boldsymbol{IM}}$ are shown. Candidate hub genes are marked by an ‘*’ beside them.

| Hub Gene | R7 KME | B8 KME | K9 KME | Mean KME | t-test p-value |
| --- | --- | --- | --- | --- | --- |
| Ppp4r2 | 0.74 | 0.83 | 0.48 | 0.68 | 0.02 |
| Tmf1 | 0.48 | 0.80 | 0.76 | 0.68 | 0.02 |
| Klhl7 | 0.25 | 0.99 | 0.76 | 0.67 | 0.09 |
| Tmem30a | 0.76 | 0.46 | 0.77 | 0.66 | 0.02 |
| Xpr1 | 0.84 | 0.71 | 0.39 | 0.65 | 0.04 |
| Dnm1l | 0.94 | 0.31 | 0.68 | 0.64 | 0.07 |
| Papola | 0.58 | 0.71 | 0.62 | 0.63 | 0.00 |
| Cntn1* | 0.86 | 0.20 | 0.81 | 0.62 | 0.10 |
| Mapk1* | 0.96 | 0.32 | 0.53 | 0.60 | 0.08 |
| Pafah1b2 | 0.73 | 0.55 | 0.49 | 0.59 | 0.01 |
| Gnai1 | 0.94 | 0.26 | 0.55 | 0.58 | 0.10 |
| Kdm1b | 0.31 | 0.73 | 0.69 | 0.57 | 0.05 |
| Nlgn1 | 0.59 | 0.58 | 0.51 | 0.56 | 0.00 |
| Tm2d1 | 0.32 | 0.59 | 0.75 | 0.56 | 0.05 |
| Gpm6b | 0.64 | 0.38 | 0.55 | 0.52 | 0.02 |
| Tardbp | 0.50 | 0.75 | 0.31 | 0.52 | 0.05 |
| Fbxo8 | 0.56 | 0.38 | 0.53 | 0.49 | 0.01 |
| Prepl | 0.96 | 0.24 | 0.26 | 0.49 | 0.18 |
| Pggt1b | 0.84 | 0.21 | 0.40 | 0.48 | 0.12 |
| Rragd | 0.63 | 0.55 | 0.27 | 0.48 | 0.05 |

Supplementary Table S9. Repeatability of aged R7 yellow module hub genes in B8 aged matching (red and brown) modules. Twenty hub genes with the highest mean $\boldsymbol{k}_{\boldsymbol{IM}}$ are shown. Candidate hub genes are marked by an ‘*’ beside them.

| Hub Gene | R7 KME | B8 KME | Mean KME | t-test p-value |
| --- | --- | --- | --- | --- |
| Stxbp1* | 0.95 | 0.70 | 0.83 | 0.10 |
| Dlgap1* | 0.75 | 0.86 | 0.81 | 0.04 |
| Psmd8 | 0.59 | 0.93 | 0.76 | 0.14 |
| Nsf | 0.90 | 0.58 | 0.74 | 0.13 |
| Dpp6* | 0.94 | 0.48 | 0.71 | 0.20 |
| Glul | 0.75 | 0.67 | 0.71 | 0.04 |
| Zfp706 | 0.61 | 0.76 | 0.69 | 0.07 |
| Tmem30a | 0.82 | 0.54 | 0.68 | 0.13 |
| Ptk2b | 0.56 | 0.75 | 0.66 | 0.10 |
| Gpm6b | 0.34 | 0.92 | 0.63 | 0.27 |
| Zfp238 | 0.77 | 0.48 | 0.63 | 0.14 |
| Rab3a | 0.61 | 0.65 | 0.63 | 0.02 |
| Rac1 | 0.77 | 0.49 | 0.63 | 0.14 |
| Ube2l3 | 0.69 | 0.57 | 0.63 | 0.06 |
| Thy1 | 0.67 | 0.57 | 0.62 | 0.05 |
| Rnf4 | 0.85 | 0.37 | 0.61 | 0.24 |
| Trim9 | 0.66 | 0.56 | 0.61 | 0.05 |
| Skp1 | 0.52 | 0.68 | 0.60 | 0.08 |
| Vps52 | 0.63 | 0.56 | 0.60 | 0.04 |
| Pkia | 0.62 | 0.56 | 0.59 | 0.04 |

Supplementary Table S10. Repeatability of aged R7 yellow module hub genes in B7 aged matching (purple and yellow) modules. Twenty hub genes with the highest mean $\boldsymbol{k}_{\boldsymbol{IM}}$ are shown. Candidate hub genes are marked by an ‘*’ beside them.

| Hub Gene | R7 KME | B7 KME | Mean KME | t-test p-value |
| --- | --- | --- | --- | --- |
| Nek9 | 0.77 | 0.82 | 0.79 | 0.02 |
| Il1rap | 0.79 | 0.77 | 0.78 | 0.01 |
| Lyst | 0.83 | 0.69 | 0.76 | 0.06 |
| Prkacb* | 1.00 | 0.45 | 0.72 | 0.23 |
| Kit | 0.94 | 0.46 | 0.70 | 0.21 |
| Camk1g* | 0.70 | 0.70 | 0.70 | 0.00 |
| Gpam | 0.71 | 0.67 | 0.69 | 0.02 |
| Lgr4 | 0.75 | 0.62 | 0.68 | 0.06 |
| B3gat1 | 0.77 | 0.58 | 0.67 | 0.09 |
| Mapk1* | 0.94 | 0.40 | 0.67 | 0.24 |
| Akap1 | 0.67 | 0.66 | 0.67 | 0.00 |
| Zfp706 | 0.61 | 0.72 | 0.67 | 0.05 |
| Gabbr1 | 0.83 | 0.49 | 0.66 | 0.16 |
| Atp1b2 | 0.75 | 0.55 | 0.65 | 0.09 |
| Grm7 | 0.78 | 0.51 | 0.65 | 0.13 |
| Kcnq3 | 0.78 | 0.51 | 0.65 | 0.13 |
| Bmp3 | 0.55 | 0.70 | 0.62 | 0.08 |
| Bcl2l1 | 0.71 | 0.54 | 0.62 | 0.09 |
| Nlgn3 | 0.73 | 0.51 | 0.62 | 0.11 |
| Tef | 0.87 | 0.37 | 0.62 | 0.24 |

Supplementary Table S11. Repeatability of aged R7 yellow module hub genes in B7 aged matching (purple and yellow) modules and aged B8 matching (brown and yellow) modules. Hub genes with the highest mean $\boldsymbol{k}_{\boldsymbol{IM}}$ are shown. Candidate hub genes are marked by an ‘*’ beside them. There were only five hub genes that were common among the three networks.

| Hub Gene | R7 KME | B7 KME | B8 KME | Mean KME | t-test p-value |
| --- | --- | --- | --- | --- | --- |
| Zfp706 | 0.61 | 0.72 | 0.76 | 0.70 | 0.00 |
| Dlgap1* | 0.75 | 0.43 | 0.86 | 0.68 | 0.03 |
| Sqstm1 | 0.46 | 0.70 | 0.31 | 0.49 | 0.05 |
| Grb2 | 0.23 | 0.43 | 0.52 | 0.39 | 0.04 |
| Zfp386 | 0.22 | 0.23 | 0.52 | 0.32 | 0.08 |
